# T cell activation and differentiation is modulated by a CD6 domain 1 antibody Itolizumab

**DOI:** 10.1371/journal.pone.0180088

**Published:** 2017-07-03

**Authors:** Usha Bughani, Arindam Saha, Anshu Kuriakose, Reshmi Nair, Ravindra B. Sadashivarao, Rasika Venkataraman, Swati Patel, Anuja Tushar Deshchougule, Satish Kumar S., Enrique Montero, Harish V. Pai, Dinesh V. Palanivelu, Ramakrishnan Melarkode, Pradip Nair

**Affiliations:** Research and Development, Biocon Research Limited, Bangalore, India; Radboud university medical center, NETHERLANDS

## Abstract

CD6 is associated with T-cell modulation and is implicated in several autoimmune diseases. We previously demonstrated that Itolizumab, a CD6 domain 1 (CD6D1) specific humanized monoclonal antibody, inhibited the proliferation and cytokine production by T lymphocytes stimulated with anti-CD3 antibody or when co-stimulated with ALCAM. Aberrant IL-17 producing CD4^+^ helper T-cells (Th17) have been identified as pivotal for the pathogenesis of certain inflammatory autoimmune disorders, including psoriasis. Itolizumab has demonstrated efficacy in human diseases known to have an IL-17 driven pathogenesis. Here, in *in vitro* experiments we show that by day 3 of human PBMC activation using anti-CD3 and anti-CD28 co-stimulation in a Th17 polarizing milieu, 15–35% of CD4^+^ T-cells overexpress CD6 and there is an establishment of differentiated Th17 cells. Addition of Itolizumab reduces the activation and differentiation of T cells to Th17 cells and decreases production of IL-17. These effects are associated with the reduction of key transcription factors pSTAT3 and RORγT. Further, transcription analysis studies in these conditions indicate that Itolizumab suppressed T cell activation by primarily reducing cell cycle, DNA transcription and translation associated genes. To understand the mechanism of this inhibition, we evaluated the effect of this anti-human CD6D1 mAb on ALCAM-CD6 as well as TCR-mediated T cell activation. We show that Itolizumab but not its F(ab’)2 fragment directly inhibits CD6 receptor hyper-phosphorylation and leads to subsequent decrease in associated ZAP70 kinase and docking protein SLP76. Since Itolizumab binds to CD6 expressed only on human and chimpanzee, we developed an antibody binding specifically to mouse CD6D1. This antibody successfully ameliorated the incidence of experimental autoimmune encephalitis in the mice model. These results position CD6 as a key molecule in sustaining the activation and differentiation of T cells and an important target for modulating autoimmune diseases.

## Introduction

CD6 lymphocyte surface receptor is an accessory molecule involved in the modulation of certain immune cellular processes such as thymocyte maturation and peripheral T-cell activation [1–8]. This receptor, expressed on peripheral blood T lymphocytes, medullary thymocytes and the B1 subset of B cells, has recently been identified as a target for the treatment of various autoimmune/inflammatory diseases such as psoriasis, multiple sclerosis (MS), rheumatoid arthritis (RA), and Sjogren’s disease [6–15]. Additionally, activated leukocyte cell adhesion molecule (ALCAM/CD166), the known ligand for CD6 domain 3, is over-expressed in blood brain barrier cells within the central nervous system of MS patients and in experimental autoimmune encephalomyelitis lesions [16]. Similar increased expression of the CD6 ligand is also observed in RA and Sjogren’s disease tissues (epithelial cells) as well as in activated T-cells [17, 18].

IL-17 producing CD4^+^ helper T-cells (Th17) have been identified as pivotal for the pathogenesis of certain inflammatory disorders. Pathogenic Th17 cells, recently characterized as dual IFN-γ and IL-17 expressing cells, have been implicated in psoriasis, type 1 diabetes, MS, and other autoimmune diseases [19, 20]. In recent clinical trials, antibodies targeting IL-17A and IL-17Ra have demonstrated remarkable efficacy in psoriasis patients [21–25]. Also, the pathogenic potential of Th17 cells has been identified in MS [19, 26]. Interestingly, clones of Th17 cells derived from MS patients were found to overexpress CD6 [19, 20]. Relevant to the current studies, CD6 and CD5, both being members of the scavenger receptor cysteine rich domain superfamily (SRCR-SF) and sharing considerable structural and functional homology, were individually found to be superior than classical CD28 mediated co-stimulation with anti-CD3 to prime naïve T-cells to differentiate into Th17 cells [27].

Itolizumab is a humanized IgG1 non-depleting monoclonal antibody (mAb) which binds to domain 1 of CD6 without interfering with ALCAM and CD6 domain 3 interaction [28]. Recent clinical trials with Itolizumab have demonstrated efficacy in psoriasis and rheumatoid arthritis patients, and this drug is now approved for treatment of psoriasis in India [29–32]. However, the mode of action of this drug is not clearly understood.

In our earlier study, we have shown the impact of Itolizumab on human Th1 cells [33]. In this study, we demonstrate that even under the classical co-stimulation by anti-CD3 and anti-CD28 [1, 34–38], Itolizumab is able to down-regulate the expression of key Th17 determining transcription factors and effector cytokines (i.e., IL-17) in addition to decreasing Th1 effector cytokine (IFN-γ).

Our results further establish an interesting mechanism of action of this anti-CD6 mAb on human lymphocytes, which involves reduction in CD6 phosphorylation and associated signaling molecules leading to decreased T cell activation and differentiation. While, the clinical efficacy of Itolizumab has been proven in psoriasis and RA [15, 31, 39], in this study we report for the first time the effect of an anti CD6 domain 1 murine specific antibody in ameliorating the incidence of EAE in a mice model of the disease, Multiple Sclerosis. Therefore, our results point to an immunomodulatory role for Itolizumab and establishes the relevance of targeting CD6, to regulate autoimmune disorders.

## Materials and methods

### Antibodies and reagents

All monoclonal antibodies, Itolizumab, Nimotuzumab (humanized anti-EGFR, identical Fc region as Itolizumab) and anti-mouse CD6 domain 1 (SRCR1) (m CD6D1 mAb) were produced at Biocon Ltd (Bangalore, India) and used in soluble form in all the experiments. Nimotuzumab, was used as a non-specific isotype control antibody in all human experiments (Iso Ab). Mouse anti-human CD6 FITC (MEM-98 clone, MCA1880F), CD3-FITC (MEM-57 clone, MCA2184F), CD4-Alexa Fluor® 647 (RPA-T4 clone, MCA1267A647), CD8 Alexa Fluor® 647 (MCA-1226A647) and CD25-FITC (MEM-181 clone, MCA2127F) were procured from AbD Serotec (Kidlington, Oxfordshire, UK). Mouse anti-human CD196 (CCR6)-PE (559562), RORγT-PE (563081), IL-17A-Alexa Fluor® 647 (560437), CD25 FITC (555431), CD3-Alexa Fluor® 647 (555335), PE Annexin V Apoptosis Detection Kit 1 (559763) were obtained from BD Pharmingen™ (Franklin Lakes, New Jersey, USA). Antibodies against Phospho-Stat3 (Tyr705) (D3A7) XP™ Rabbit mAb- Alexa Fluor® 488 Conjugate (4323), phospho-Tyr (9411), Zap 709 (3165), SLP76 (4958), phospho SHP1 (8849), SHP1 (3759), phospho SHP2 (3705), SHP2 (3752) were obtained from Cell Signaling Technology (Denver, MA, USA). Anti-human CD3 (OKT3 clone, 16-0037-85), Human Fc receptor binding inhibitor (16–9161) was procured from eBioscience (San Diego, CA, USA). Rabbit serum was procured from Bioneeds, Bangalore. Purified mouse anti-human IFN-γ (554699), Purified NA/LE mouse anti-human IFN-γ (554698), purified NA/LE rat anti-human IL-4 (554481) and recombinant human IL-6 (550071), transforming growth factor-beta (TGF-β), were obtained from BD biosciences (354039) (Franklin Lakes, New Jersey, USA). TGF-β was also procured from stem cell technology (02847) (Vancouver, British Columbia, Canada). Recombinant human interleukin–1β (PHC0811) and recombinant human IL-23 (PHC9321) were obtained from Invitrogen, Life Technologies^TM^ (Carlsbad, CA, USA). Recombinant Human ALCAM/CD166 Fc Chimera (656-AL) was obtained from R&D systems (Minneapolis, MN 55413, USA). T-cell activation/expansion Kit, human (130-091-441), obtained from Miltenyi Biotec GmbH, (Bergisch Gladbach, Germany), was used to prepare anti-CD3 and anti-CD28 coated beads as per manufacturer’s protocol. Phorbol 12-myristate 13-acetate (PMA) (P8139) and ionomycin (I9657) were obtained from Sigma Aldrich (St. Louis, MO, USA). BD Golgi Plug^TM^ (555029) and BD Cytofix/Cytoperm™ fixation/permeabilization buffer (554722) were obtained from BD Biosciences. Rainbow calibration 8 peaks beads (RCP-30-5A) for receptor density calculation were obtained from Spherotech, (Lake Forest, IL, USA). Biotinylated Itolizumab for CD6 detection was custom generated at Abexome Biosciences, (Bangalore, India). m CD6D1 mAb producing rat-mouse hybridoma cells were received from CIM (Center for molecular immunology, Cuba, Havana). Cells were maintained in serum free media and used for production of m CD6D1 mAb. Purification of antibody from cell supernatant was done as standard routine purification of rat antibodies. Purified antibodies were qualified for purity and integrity using reducing, non-reducing gel electrophoresis, ELISA, Flow cytometry methods and Biacore Binding data. Rat mouse MOG _35–55_ peptide, Incomplete Freund’s adjuvant and pertussis toxin and goat anti-human IgG FITC, Fc specific (F9512) were purchased from Sigma Aldrich (St. Louis, MO.). Mycobacterium tuberculosis Heat inactivated H37Ra was purchased from Difco (Fisher, Pittsburgh, PA). Isotype control rat IgG (012-000-003) antibodies (m Iso Ab) were ordered from Jackson immunoresearch (Bar Harbor, ME). Anti-mouse CD3 (16-0031-86) used in culture were purchased from e-Biosciences (Dorset, UK). Cytometeric bead array (CBA) inflammatory cytokines kit (Interleukin-6, Interleukin-10, Interferon-γ, Tumor Necrosis Factor-α) and Interleukin-17A flex set assay kit were purchased from BD Biosciences (San Jose, CA).

### Animals studies

8–12 week old female C57BL/6 mice were obtained from Harlan (Jerusalem, Israel) and maintained in the animal care facility under standard pathogen free conditions (Syngene international Ltd, Bangalore, India). The animal handling and procedures were approved and are in accordance with the guidelines from Institutional Animal Ethics Committee (Reg.No. 1089/bc/CPCSEA dt 17/09/2009), Syngene International, Bangalore, India.

#### EAE disease induction and antibody dosing

Disease was induced in mice following a study procedure described previously [40]. Briefly, EAE was induced with MOG_35–55_ emulsified with Complete Freund’s Adjuvant (CFA) and pertussis toxin. Randomized mice were injected with 60 μg in 100 μl per dose of m Iso Ab or m CD6D1 mAb from day 15 to day 27. Drugs were dosed intraperitoneally (IP). This dose translates to around 1.6mg/kg human equivalent Dose (HED) used in the clinic every alternate week. EAE clinical score was evaluated on a 5 point scale as EAE Grade [40] for all three groups (m Iso Ab and m CD6D1 mAb treated EAE-induced animals, third group being the naïve mice as no disease control). All animal were sacrificed at the end of the study as per IEAC.

#### Histopathology of spinal cord

Spinal cords were collected in 10% buffered formalin from the three groups of animals sacrificed, and described earlier. Briefly, the tissues were processed and cut longitudinally at 5μm thickness. One set of slides was stained with H&E staining for screening of inflammation. The other set was stained with Luxol fast blue staining for screening of demyelination in spinal cord. Thoracic and lumbar segments were considered for inflammation and demyelination in spinal cord of mice. Photographs were taken with Nikon 80i fitted with DFC420 camera (Leica) Images were captured with Leica Analysis Suite (LAS) 40X magnification.

#### *Ex vivo* experiments with mouse splenocytes

Splenocytes (0.2x10^6^cells/well) from three groups were stimulated with pre-coated anti-CD3 mAb (0.25 μg/well) or MOG specific antigen (20 μg/ml) in U bottom 96 well plates (Nunc). Splenocytes in uncoated wells were used as un-stimulated control cells. On day 3, supernatants were collected and cytokine measurement was performed using cytometeric bead array (CBA) inflammatory cytokine kit as per manufacturer's instructions. Data was acquired in flow cytometer CyAn ADP (Beckman Coulter, Fullerton, USA) and in FACSVerse (analyzed using FCAP array V3. On day 4 of similar experiments in parallel plates, T cell proliferation was measured using Alamar Blue reagent.

### Human PBMC culture for Th17 polarization

Human PBMC derived from different donors were isolated as explained previously [33]. In addition to freshly sourced PBMCs we have also used frozen PBMCs procured from Precision for Medicine Frederick, MD, USA. These frozen PBMCs were used as per vendor recommended protocol. In our hands, freshly isolated and frozen PBMCs responded similarly to T cell activation stimulus and subsequent inhibition of this activation by Itolizumab. PBMCs were counted and plated in 96 well flat bottom plates, at a density of 0.1 million cells per well and stimulated with anti-CD3 and anti-CD28 coated beads (0.05 million beads for 0.1 million cells per well) in non-polarizing (Thnp) and Th17 polarizing conditions (Th17pol). In some experiments as mentioned in the legends, soluble anti CD3 (OKT3) at 0.1 ng/ml (eBioscience 16003785) of anti-CD3 along with soluble anti-CD28 at 10 ng/ml (BD pharmingen 555725) was used for stimulation. In some PBMC proliferation experiments soluble anti CD3 (OKT3) at 0.5 ng/ml was used as stimulating agents as indicated in figure legend. Assay medium was used as described [33]. In Th17pol conditions, polarizing cytokines and growth factors were added at a final concentration of: IL-1β 10 ng/mL, IL-6 10 ng/mL, TGF-β 15 ng/mL, IL-23 10 ng/mL, anti-IL-4 10 μg/mL and anti-IFN-γ 2.5 μg/mL [27]. Itolizumab or Iso Ab were added to cells in both Thnp and Th17pol conditions at a final concentration of 40 μg/mL or at 10 μg/ml as described in the appropriate figure legends. PBMCs were cultured at 37^°^C and cells and supernatant were collected at various time points (day 3, day 6/7, day 8/9/10 and day 13/14) for flow cytometry and cytokine analysis respectively. For cells that were continuously cultured, 50 μL medium was replaced with RPMI complete medium containing 2 ng/mL recombinant human IL-2 (Invitrogen PHC0023 and R&D systems 202-IL) for Thnp condition and 2 ng/mL IL-2 along with10 ng/mL IL-23 for Th17pol condition on days 6 and 10.

### CFSE labelling and T-cell proliferation assay

PBMCs were incubated with CFSE (Invitrogen C34554 Molecular Probes, Inc. 29851 Willow Creek Road Eugene, OR 97402–9132) dye at a final concentration of 5μM, incubated at 37^°^C, CO_2_ incubator for 15 minutes (with intermittent shaking), centrifuged and resuspended in complete medium and again incubated at 37^°^C, CO_2_ incubator for 30 minutes for the stabilization. Cells were washed and stimulated in Thnp and Th17pol conditions in presence of Iso Ab or Itolizumab. On day 3 post-stimulation cells were analyzed for CFSE dilution on CD4^+^ T cells.

### Cytokine analysis of human PBMC

At each time point, 100μL of cell supernatant was collected, pooled from quadruplicate wells and frozen at -80^°^C. Cytokine analysis was performed as per manufacturer instructions using human IFN-γ Quantikine SixPak (SIF50) and human IL-17 (IL-17A) Quantikine SixPak (S1700) kits from R&D Systems (Minneapolis, MN, USA). Absorbance was read at 450/570 nm using SpectraMax M5^e^ reader, Molecular Devices, Sunnyvale, CA, USA. Concentration was calculated using the standard provided along with the kits.

### Flow cytometry analysis

#### Cell surface markers, activation markers, Annexin V-7AAD staining

At each time point, cells from quadruplicate wells were harvested and blocked with human Fc receptor binding inhibitor by incubating at 4^°^C for 30 minutes with appropriate dilution of Fc blocking reagent. In some experiments, incubation at room temperature for 30 minutes with 10% rabbit serum was used for blocking Fc receptors. Cells were centrifuged and re-suspended in staining buffer (2% FBS in 1X PBS) containing the antibodies for cell surface markers. For Annexin V-7AAD staining buffers available in the kits and protocol for staining was followed as per manufacturer suggestion.

#### Intracellular staining for pSTAT3

After surface marker (CD4) staining, pSTAT3 staining was performed as described previously [33].

#### Intracellular cytokine staining for IL-17A, IFN-γ and transcription factor RORγT/pSTAT3

At the time point of analysis, cells were left unstimulated or stimulated with 50 ng/mL of PMA and 1 μg/mL of ionomycin in presence of BD Golgi Plug^TM^ and incubated for 5 hours at 37^°^C. Cells were harvested and washed in staining buffer and blocked with human Fc receptor binding inhibitor. After surface marker staining, cells were re-suspended in BD Cytofix/Cytoperm™ fixation/permeabilization buffer, and intracellular staining was performed as per manufacturer’s instructions.

Acquisition and analysis of samples were performed using a CyAn-ADP flow cytometer with the Summit version 4.3 software (Beckman Coulter, Fullerton, CA, USA). For some experiments, acquisition and analysis of samples was performed using a BD FACSVerse flow cytometer with BD FACSuite version 1.0.5. Lymphocytes were gated by forward and side scatter and further gated on CD4^+^ or CD8^+^ or CD3^+^ T-cells as mentioned in the respective experiments. In some experiments, total cells were selected and gated to get uniform event count display, as mentioned in respective figure legends.

For calculation of number of CD6 molecules per cell (receptor density), spherotech 8 peak beads were used. The analysis is same as described previously [33]. Briefly, 8 peak beads were acquired at the same voltage settings as the other samples. FL2 (PE Channel) was used to calculate expression of CD6 molecules/cell on CD4^+^ and CD8^+^ T-cells.

### Microarray analysis

The experimental protocol as explained in the section on human PBMC culture for Th17 polarization was followed, and cells from multiple wells were harvested at different time points and snap frozen in liquid nitrogen. A parallel plate in the same experimental setup was used to confirm Th17 polarization. Later, RNA was isolated from these samples and microarray analysis was performed at Genotypic Technology (P) Ltd. using Agilent human gene expression custom 8X15K (15208 genes) array(AMADID: 16332) designed by Genotypic Technology Private Limited. The detailed methodology for microarray analysis is described previously [33].

#### Feature extraction

Data extraction from Images was done using Agilent Feature Extraction software Version 11.5.

#### Microarray data analysis

Images were quantified using Agilent Feature Extraction Software. Feature extracted raw data was analysed using Agilent GeneSpring GX Version 12.0 software. Normalization of the data for cells treated with isotype control mAb and cells treated with Itolizumab in Th17pol conditions was done against the unstimulated cells (Day1) in GeneSpring GX using the 75^th^ percentile shift method (percentile shift normalization is a global normalization, where the locations of all the spot intensities in an array are adjusted). The day wise conditions of control and Itolizumab group were further categorized into early (Day3), middle (Day6 and Day8) and late (Day10 and Day14) stages. Differential expression patterns were identified for early, middle & late stages with ≥2 fold cut-off, (log_2_1) in control mAb treated cells over unstimulated cells. Further these probes were identified in Itolizumab group. The scatter plots were generated for early, middle & late stages, for the probes showing a minimum 20% reduction in expression in Itolizumab when compared to Iso Ab group. Regression lines and R-Squared value were obtained for these probes using the linear model fit from R package. Gene ontology functional classification was performed using DAVID database tool for the genes in the Itolizumab group, reduced by at least 20% relative to control group. Significant gene ontology terms were determined using Fisher Exact P-Value. The accession number for the microarray data is GSE64382 and data set is submitted to Geo Gene Expression Omnibus.

### F(ab’)2 generation from Itolizumab

The F(ab’)2 fragment of Itolizumab was generated using Pierce F(ab’)2 preparation kit (Cat. No. 44988) from Thermo Scientific (Rockford, IL) as per manufacturer’s instruction and also commercially procured from Bioneeds Inc. (Bangalore, India). The F(ab’)2 fragment was characterised by its molecular weight (as shown in reducing and non-reducing gel) and equivalent binding affinity to human CD6 domain 1 compared to full length Itolizumab.

### CD6 phosphorylation assay

For ALCAM-CD6 interaction experiments, 6 well plates were coated overnight with Fc-ALCAM (10 μg/ml) in TSM buffer (20 mM Tris, 150 mM NaCl, 1 mM CaCl_2_, 2 mM MgCl_2_, 1X protease and phosphatase inhibitors added). On the day of experiment coated plates were blocked with 1% BSA in TSM buffer. Human PBMC (5x10^6^/well in a 6 well plate) were plated and treated with different conditions for 40 minutes [1]. In case of TCR experiments, PBMC were treated with 0.5 ng/ml anti-CD3 antibody (OKT 3) for 24 h in presence or absence of Itolizumab or Iso antibody. Cells were harvested and CD6 was immune-precipitated with Itolizumab or Iso Ab. 10% total lysate served as input control samples. Both immune precipitated and input control samples were immune blotted and analyzed.

### Immuno-blot

Immuno blot from co-immuno precipitated as well as 10% input control samples were done as described earlier [41]. The x-ray films were scanned and quantified using Image J software [42]. The bar graphs from the quantified images were made in Graph Pad prism 7.0 software.

### Biacore assays

All Biacore reagents were procured from GE Healthcare, the anti-His antibody was from Qiagen. Briefly the CM5 chips were immobilized with anti-His antibody (1000RUs) using standard amine coupling procedure. The full length CD6 ligand was captured at 5 μg/mL in HBS-P running buffer. A concentration series of each of the analyte (m CD6D1 mAb, Itolizumab and F(ab’)2 fragment of Itolizumab respectively) was injected at a flow rate of 50 μL/min at 25°C. The association and dissociation phases were monitored for 250s and 600s respectively. The surface was regenerated using 10mM Glycine HCl, pH 1.5. The ligand-analyte interaction was obtained by fitting the response data in 1:1 Langmuir fit using BIA-evaluation software.

### Statistical analyses

GraphPad Prism 7.0 software (GraphPad software, San Diego, CA, USA) was used for all statistical analyses. P values ≤ 0.05 were considered significant for all experiments. Details of specific statistical tests used are provided in the respective figure legends.

### Ethics statement

The research program in human subjects was approved by the Independent Ethics Committee Consultants at Syngene International (Protocol No. CLCD-039-16). Human PBMCs were obtained from healthy volunteer blood donors after signing an informed consent form. The animal studies were approved by Institutional Animal Ethics Committee (IAEC) at Syngene International Limited.

## Results

### Increased expression of CD6 on T cells after activation in Th17 polarizing conditions

Naïve Human T-cells can be differentiated *in vitro* into Th1, Th2 or Th17 phenotype based on the context of the cytokine milieu they receive [27, 43–46]. IL-6 and TGF-β induce differentiation of naïve T-cells into Th17 subset and this strategy was used to generate Th17 cells *ex vivo* from human PBMCs.

**Fig 1:**
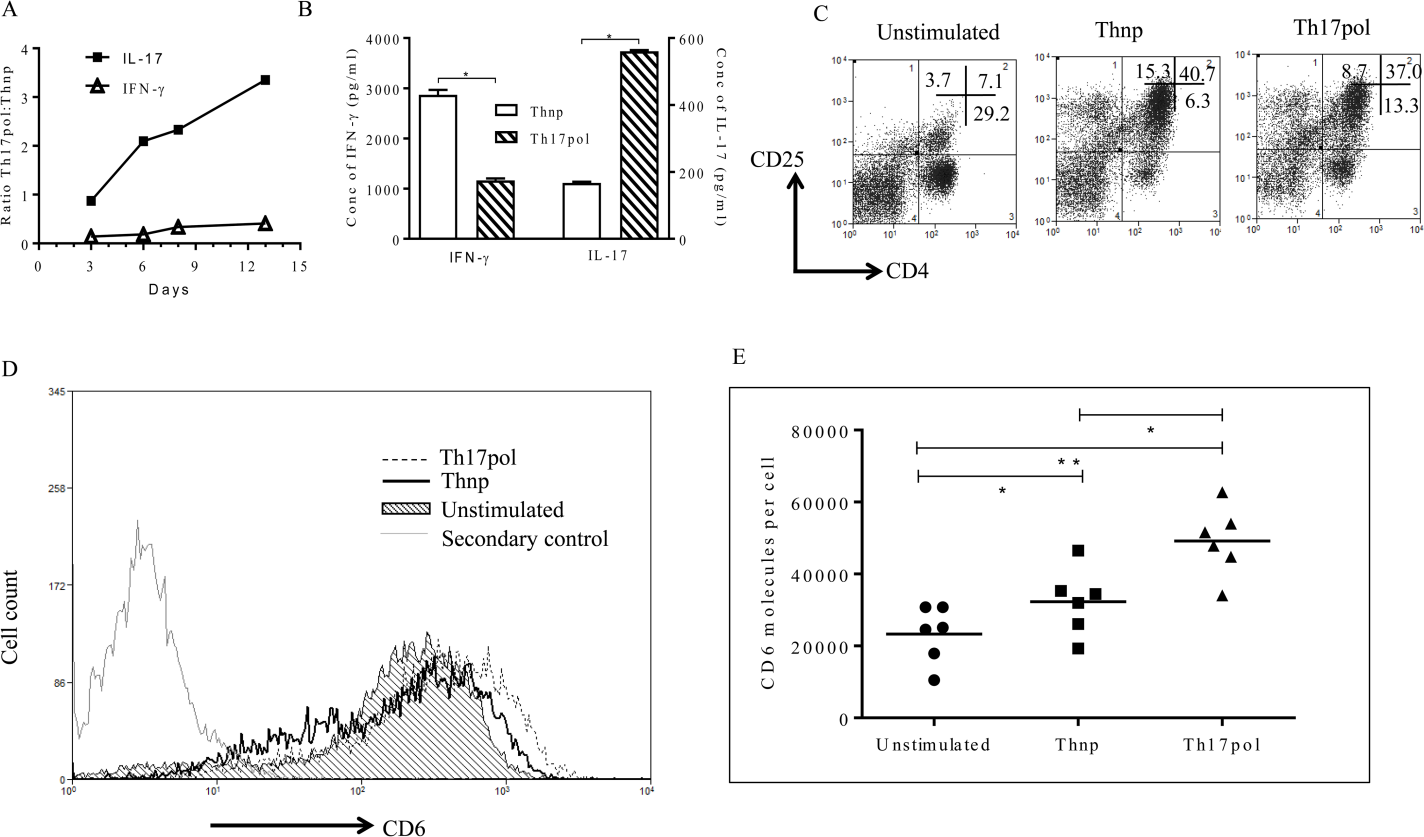
Increased expression of CD6 on T cells after activation in Th17 polarizing conditions. (A) PBMCs were stimulated in Thnp or Th17pol conditions, supernatant was collected and analyzed for secreted IFN-γ (Th1 signature cytokine) and IL-17 (Th17 signature cytokine). Ratio of absolute concentration of IFN-γ and IL-17 in Th17pol and Thnp conditions (Th17pol: Thnp) is plotted across the days of analysis. (B) Absolute levels of IFN-γ and IL-17 on day 13 is compared between Thnp and Th17pol conditions. Data shown is mean ±SD for triplicate ELISA wells (*p≤0.05). (C) PBMCs were left unstimulated or stimulated in Thnp or Th17pol conditions. CD25 expression on CD4^+^ T cells was analyzed on Day 3. Percentage of cells are indicated in the quadrants. Dot plots are gated on lymphocyte scatter. (D) PBMCs were left unstimulated (shaded histogram) or stimulated in Thnp or Th17pol conditions. CD6 expression (using biotinylated Itolizumab as detection reagent) was analyzed on Day 9 and plotted as CD6 overlay histograms gated on lymphocyte scatter. The secondary alone histogram is also overlayed for reference. (E) CD6 molecules/cell (receptor density) in unstimulated, Thnp and Th17pol conditions on gated CD4^+^ T-cells is shown as a scatter plot using biotinylated Itolizumab as detection reagent (*p≤ 0.05). For Fig 1B and 1E, statistical significance was determined using non-parametric unpaired t-test followed by Mann-Whitney test. In panels A and B, data is representative of 3 independent similar experiments, panel C is representative from at least 3 independent experiments, panel D is representative data from 6 donors and panel E has data from 6 donors.

As shown in Fig 1A, by day 13, the increase in secreted IL-17 level was 3–4 fold higher than the rise in IFN-γ release in cells stimulated in Th17pol conditions (i.e. PBMCs stimulated with anti-CD3 and anti-CD28 beads in presence of Th17 polarizing milieu) as compared to Th-17 non-polarizing (Thnp) cells (PBMCs stimulated with anti-CD3/anti-CD28 beads without added cytokines). Absolute concentrations of IL-17 and IFN-γ on day 13 are shown in Fig 1B. IFN-γ release by cells stimulated in Th17pol condition was significantly low (around 3 fold) as compared to the Thnp cells. On the contrary, these Th17pol cells showed a 3–4 fold increase in secreted IL-17 levels as compared to Thnp cells. These results indicate that under Th17pol conditions a shift towards differentiation to Th17 cells was initiated as early as day 3 with full establishment by day 13.

T cell activation is measured by increased expression of CD25 (IL-2Rα) on CD4^+^ cells [33]. Here we show a 5–6 fold increase in CD4^+^/CD25^+^ double positive lymphocyte population over unstimulated cells in both Thnp as well as Th17pol cultured condition (Fig 1C). This suggests similar activation of T cells in both Thnp as well as Th17pol conditions.

The surface expression of CD6 receptors on T-cells increased after 48 hours of stimulation and was sustained until late into polarization. On day 9 post stimulation, biotinylated Itolizumab used as a detection reagent, identified increased CD6 expression on lymphocytes (Fig 1D). 15–30% and 25–35% of CD4^+^ T-cells in Thnp and Th17pol conditions respectively (S1 Fig). As shown in Fig 1E, Thnp and Th17pol CD4^+^ T-cells showed a significant increase in CD6 molecules/cell (receptor density) as compared to the unstimulated cells. The mean number of CD6 receptor molecules per cell were 23284, 32268, and 49151 on unstimulated, Thnp and Th17pol stimulated PBMCs respectively (gated on CD4^+^ T-cells) as plotted in Fig 1E. In addition, CD6 overexpression on CD4^+^T cells in Th17 polarizing conditions was even higher than that in Thnp conditions and this was statistically significant (Fig 1E).

The increase in CD6 expression was also confirmed with the use of other commercially available domain1 binding anti-CD6 antibodies. This increased expression was not only limited to CD4^+^ T-cells but was also observed in activated CD8^+^ T-cells (S2 Fig). In CD8^+^ T-cells, the mean values/number of CD6 receptor molecules per cell were 11149, 13019 and 15480 on unstimulated, Thnp and Th17pol stimulated PBMCs respectively (gated on CD8^+^T-cells). In addition, when specifically gated on CD25^hi^ CD4/CD25^hi^ CD8^+^ cells, the MFI for CD6 expression was highest indicating that activated T cells show enhanced CD6 expression (S1 Table).

### Itolizumab inhibits T-cell activation and proliferation in both Thnp and Th17pol conditions

**Fig 2:**
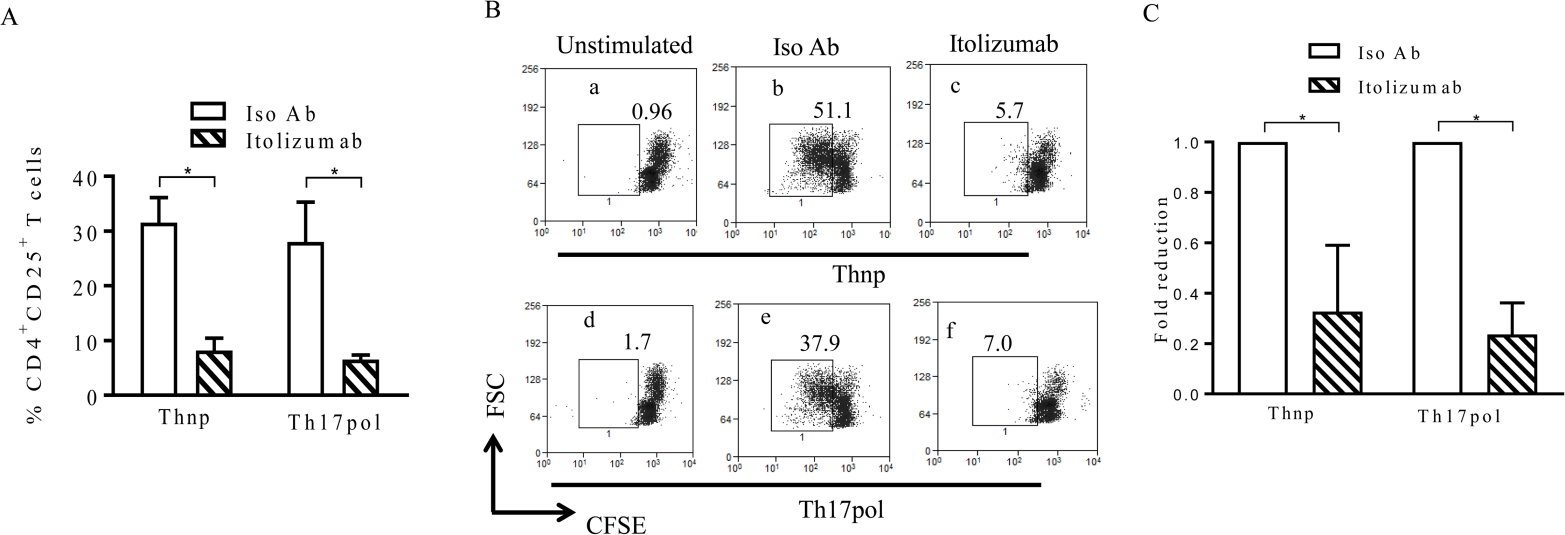
Itolizumab inhibits T-cell activation and proliferation in both Thnp and Th17pol. conditions. (A) PBMCs were stimulated in Thnp or Th17pol conditions in presence of Itolizumab or isotype control mAb (Iso Ab) both at 40 μg/ml. On day 3, cells gated on lymphocyte scatter and CD4^+^T-cells were analyzed for CD25 expression. Percent CD4^+^CD25^+^ T-cells in stimulated PBMCs, is plotted as bar graphs. Data shown is mean±SD from 3 different donors (*p≤0.05). (B) PBMCs labelled with CFSE dye were stimulated in Thnp (a-c) or Th17pol (d-f) conditions in presence of Itolizumab (c and f) or Iso Ab (b and e). On day 3, cells were analyzed for CFSE dilution on cells gated on lymphocyte scatter and CD4^+^ T-cells. Percent cells are indicated in dot plots of forward scatter (FS) vs CFSE. Data is representative from 3 independent experiments. (C) As derived from panel B, fold reduction in percentage of proliferated T-cells upon Itolizumab treatment is compared with Iso Ab. Fold reduction is determined by calculating percent total divided (proliferated) cells (with in the rectangular gate in Fig 2B) in Iso Ab or Itolizumab / percent total divided (proliferated) cells in Iso Ab. Bar graphs show mean±SD from 3 independent experiments. For Fig 2A and 2C, statistical significance was determined using non-parametric unpaired t-test followed by Mann-Whitney test.

We had previously reported a 50–55% decrease in CD25 expression on anti-CD3 as well as anti-CD3 and ALCAM co-stimulated PBMCs in the presence of Itolizumab [33]. Here, we show that, there is a significant reduction in CD25 expression on anti-CD3 and anti-CD28 stimulated CD4^+^ T-cells (~50% reduction) in Thnp and Th17pol conditions by Itolizumab (Fig 2A). This would imply that this anti-CD6 mAb is able to consistently and similarly inhibit the activation of T cell mediated via CD3 with or without ALCAM or CD28 mediated co—stimulation. Associated with the reduction of activation of T-cells, is the inhibition of proliferation of these cells even under Th17pol conditions. Significant reduction of T-cell proliferation (as indicated by CFSE dilution) was observed in cells stimulated in the presence of Itolizumab (Fig 2B and 2C).

To rule out any role of CD6 receptor internalization in the inhibition of T cell activation and proliferation observed, we examined presence of CD6 receptor on lymphocytes and occupancy of the same with Itolizumab in PBMCs stimulated in presence of Itolizumab or Iso Ab. Commercially available competing anti CD6 D1 antibody MEM98 [47] showed a positive signal in Iso Ab group (as the receptors are available to bind) and not in Itolizumab treated group (as the receptors are occupied with Itolizumab) (S3A Fig). To further confirm that loss of staining with commercial anti CD6 D1 in Itolizumab treated group (in Panel A) is not because CD6 receptor internalization, staining with fluorochrome conjugated anti-human IgG was performed under the same experimental conditions. Positive staining with anti-human IgG on Itolizumab treated cells and not in the Iso Ab group indicated that CD6 receptors were occupied with Itolizumab (human IgG) (S3B Fig). Taken together, both set of stainings confirm that CD6 receptors on lymphocytes are not internalized upon Itolizumab treatment and are occupied with Itolizumab.

After having observed a significant role for this anti-CD6 mAb in inhibiting T-cell activation and proliferation it was of interest to investigate its effect on Th17 cell activation and Th17 cell associated cytokine production.

**Fig 3:**
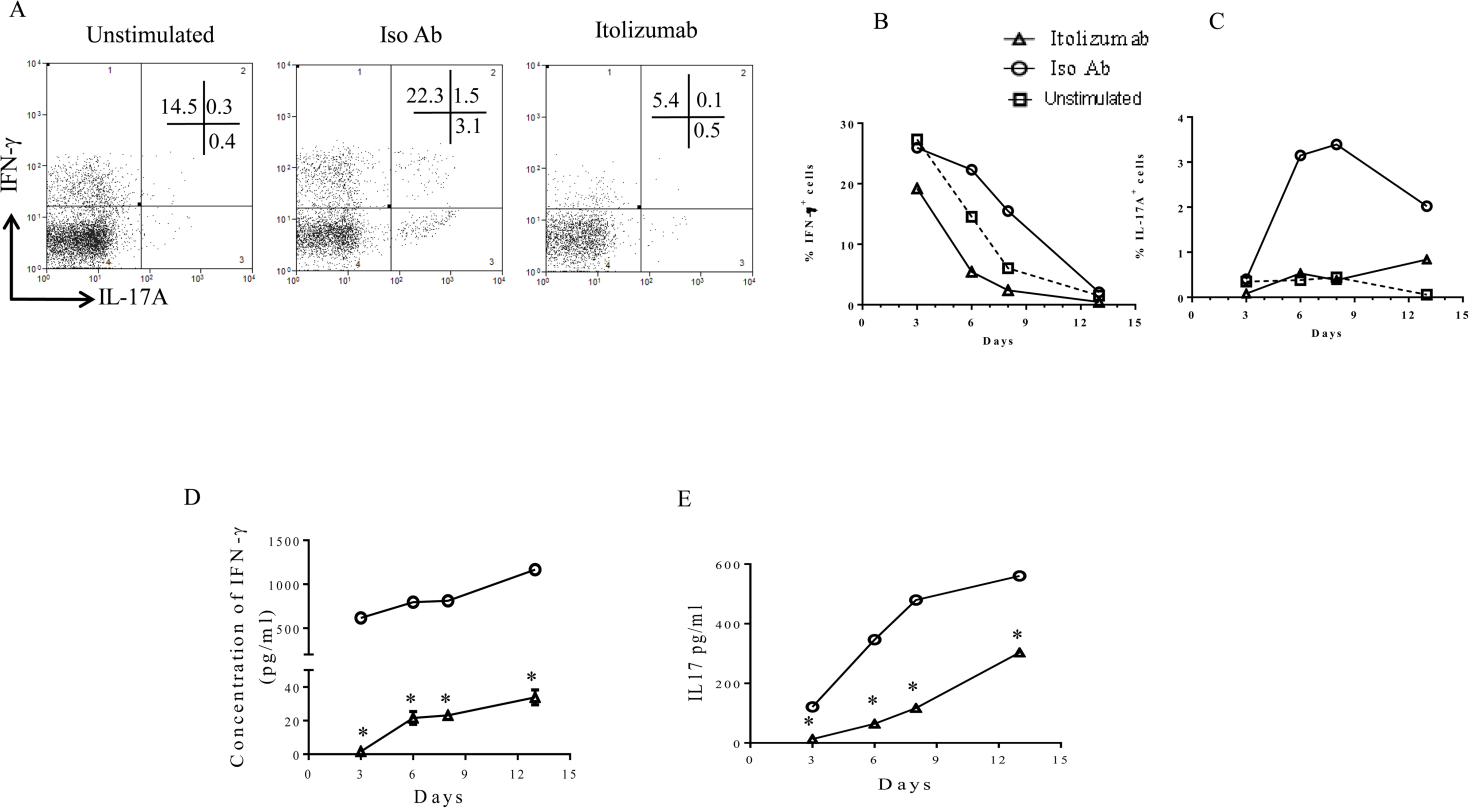
Itolizumab causes reduction in expression of IL-17 and IFN-γ cytokines in cells stimulated in Th17 polarizing conditions. PBMCs were stimulated with anti-CD3 and anti-CD28 beads in Th17pol conditions in presence of Itolizumab or Iso Ab (both at 40 μg/ml). On days 3, 6, 8 and 13 cells stimulated in Th17pol conditions with Iso Ab or Itolizumab, were re-stimulated with PMA-Ionomycin for 5 hours and analyzed for expression of intracellular cytokine IFN-γ and IL-17A. (A) Representative flow cytometry dot plots (gated on lymphocyte scatter and CD3^+^ T-cells) on day 6 are shown. Percent T-cells are indicated in the quadrants. Data is representative of 2 independent experiments. (B) Percentage of IFN-γ^+^ and (C) Percentage of IL-17A^+^ T-cells in presence of Itolizumab or Iso Ab and in unstimulated cells are plotted across days as obtained from flow cytometry analysis. Data is representative of 2 independent similar experiments. In panel/data 3A-C, before gating on lymphocyte gate, total cells were selected and gated to get uniform event count display. To analyze the level of secreted cytokines, supernatants were collected from quadruplicate wells of PBMCs stimulated in Th17pol conditions in presence of Itolizumab or Iso Ab, prior to PMA-ionomycin restimulation. As evaluated by ELISA, secreted (D) IFN-γ and (E) IL-17 levels are plotted across days. The level of cytokine release from unstimulated cells was negligible and hence is not plotted in the graphs. In Panel D and E representative data is shown as mean ± SD. Statistical analysis was performed from triplicate ELISA wells from one of the 3 independent similar experiments, for each time point in control and treated groups (*p≤ 0.05). For Fig 3D and 3E statistical significance was determined by t-test. For (B-E) open triangle, circle and box indicate Itolizumab, Iso Ab and unstimulated cells respectively.

Under Th17pol conditions intracellular expression of Th1 (IFN-γ) and Th17 (IL-17A) signature cytokines were compared between Iso Ab and Itolizumab treated cells. Representative dot plots from day 6, showed only minimal expression of both IFN-γ and IL-17A in CD3^+^ T-cells with Itolizumab treatment, as compared to Iso Ab treated groups (Fig 3A). A significant decrease in the dual IFN-γ and IL-17A positive T-cells, recently identified as pathogenic T-cells (15), was also observed (Fig 3A). Similar reductions, with Itolizumab treatment were also seen in Thnp conditions.

The time course study across days 3, 6, 8 and 13 demonstrated maximum decrease in intracellular IFN-γ on days 6 and 8 (Fig 3B). Similarly, maximum inhibition (80–90%) of intracellular IL-17A by Itolizumab was also observed on these days (Fig 3C). A corresponding pattern of decrease in IFN-γ and IL-17 release, upon Itolizumab treatment (as evaluated by measuring secreted cytokines) was also observed (Fig 3D and 3E). A similar pattern of inhibition for both the intracellular cytokines measured as well as the secreted cytokines confirmed the effect of Itolizumab in these two major effector Th subsets (Th1 and Th17 cells).

This effect of Itolizumab was also observed in CD8^+^ T-cells. Representative dot plot from day 6 sample (S4 Fig), gated on CD8^+^ T-cells show that these cells mainly secreted IFN-γ and very low IL-17A upon activation even under Th17pol conditions. Itolizumab resulted in a significant inhibition of IFN-γ secretion by these cells as compared to the control. In addition, the limited IL-17A secreted by these cells, is also inhibited in the presence of drug. Interestingly the IFN-γ and IL-17A double positive population of CD8^+^ T cells was also substantially reduced in the presence of Itolizumab.

To rule out the role of Itolizumab in inducing activation induced cell death (AICD) that might result in cytokine secretion from dying cells, cell death was evaluated using Annexin V-7AAD staining. Human PBMCs were stimulated with anti CD3 for 72 hours in presence of Itoliuzmab or Iso Ab and then stained for annexin V-7AAD. Results clearly showed that Itolizumab did not induce any AICD in activated T cells at 72 hours (S5A Fig) and this was consistent as late as 120 hours post stimulation (S5B Fig).

### Reduction in signature Th17 specific markers in the presence of Itolizumab

**Fig 4:**
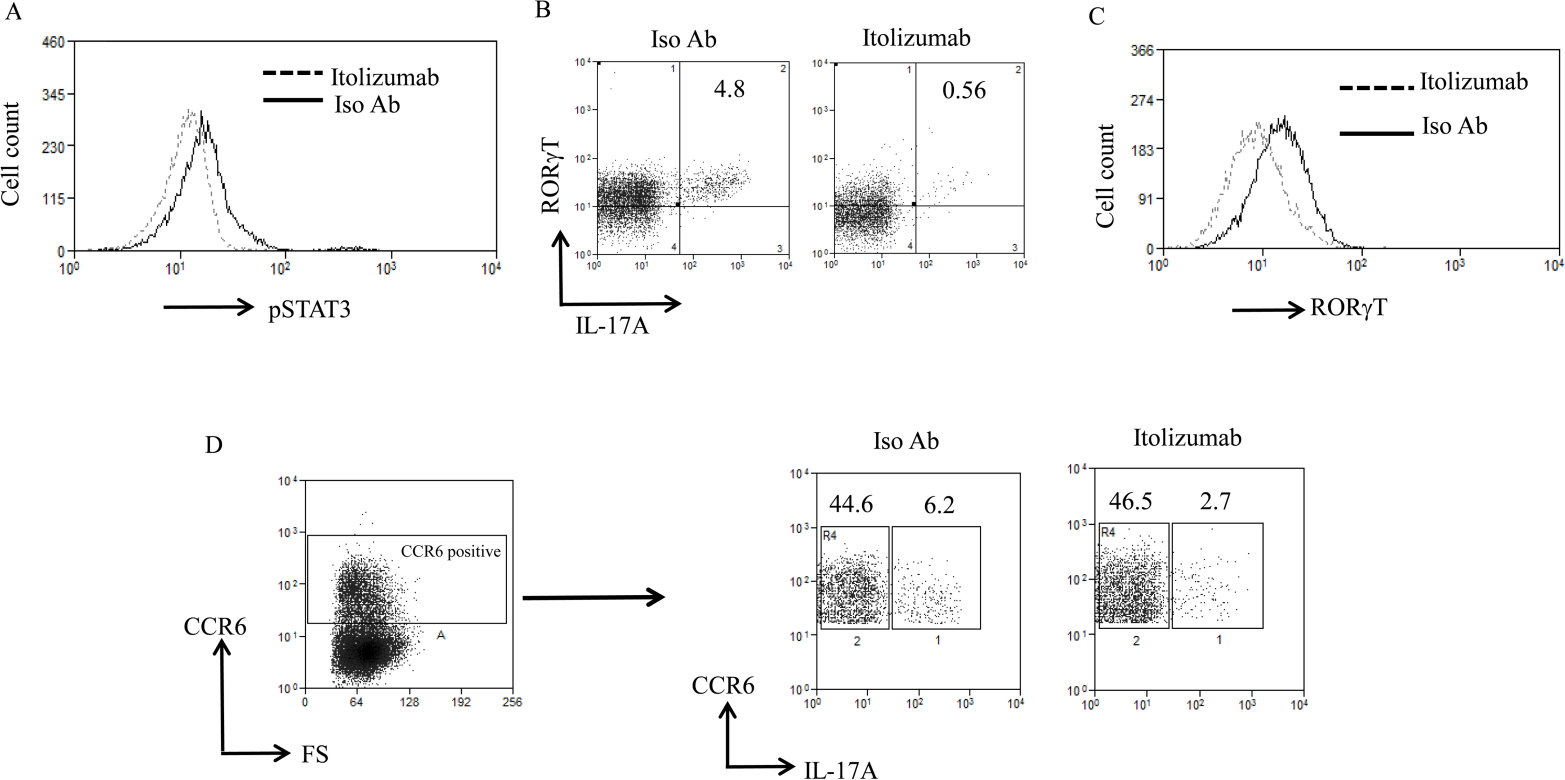
Itolizumab causes reduction in signature Th17 specific markers. **(**A) PBMCs were stimulated in Th17pol conditions in presence of Itolizumab or Iso Ab (both at 40 μg/ml) and analyzed for expression of transcription factor pSTAT3. Day 3 post stimulation data is shown as histogram for pSTAT3 on gated CD4^+^ T-cells (with a prior gating on lymphocyte scatter) and is representative of 3 independent experiments. **(**B) Cells stimulated in Th17pol condition in presence of Itolizumab or Iso Ab were re-stimulated with PMA-Ionomycin for 5 hours and analyzed for expression of intracellular cytokine IL-17A and Th17 signature transcription factor RORγT. Day 6 representative dot plots of RORγT and IL-17A gated on lymphocyte scatter and CD3^+^ T-cells are shown. Percent cells are indicated in the plots. (C) Data from panel B is plotted as histogram overlays of RORγT MFI on gated CD3^+^ T-cells stimulated in Th17pol condition in presence of Iso Ab or Itolizumab. In panel B and C, data shown is representative of 2 independent similar experiments. (D) For the experiment, similar to the one explained in panel B, Day 6 representative dot plots of CCR6 and IL-17A gated on lymphocyte scatter and CD3^+^CCR6^+^ T-cells are shown. Percent T-cells are indicated in the plots. Data is representative of 2 different time points with similar results (on Day 6 and Day 10). In panel 4B-D before gating on lymphocyte gate, total cells were selected and gated to get uniform event count display.

Th17 differentiation involves the synergism of pSTAT3 and RORγT transcription factors and they follow a sequential induction with pSTAT3 playing role upstream of RORγT [27, 43, 44, 48, 49]. Our data showed that pSTAT3 levels increased on Day1 post stimulation and were maximum at day3. In agreement with our previous study [33], Itolizumab mediated down regulation of pSTAT3 expression in CD4^+^ T cells under Th17 pol conditions. Maximum effect of Itolizumab mediated pSTAT3 downregulation was observed on day 3 post stimulation and this ranged from ~19–44% in multiple experiments (Fig 4A). Further under these polarizing conditions there was a substantial reduction in RORγT positive cells that secrete IL-17A in the presence of Itolizumab as compared to Iso Ab treated cells (Fig 4B). Correlating with the peak inhibition of effector cytokines (Fig 3B and 3C), we observed a substantial reduction in frequency of these double positive cells (70–80% reduction) (Fig 4B). Unlike pSTAT3 expression in these human PBMC experiments, RORγT expression level don’t go up substantially upon stimulation. Itolizumab was able to downregulate RORγT as evaluated at varying time points. Fig 4C is representative data from Day 6 showing reduction in expression of RORγT on CD3^+^ T cells. Similar reductions in RORγT were also observed at other time points evaluated in repeat experiments (Day 8, Day 9).

CCR6 is the characteristic marker of memory T-cells in the peripheral blood. Several reports suggest that IL-17 producing T-cells (either differentiated *in vitro* or in peripheral human blood) express CCR6 and this expression of CCR6 is a fundamental feature of Th17 differentiation [50–54]. Therefore, it was of interest to evaluate CCR6 expression in IL-17 producing T-cells and effect of anti-CD6 on them. As analyzed on day 6, in Th17pol conditions, 50–60% reduction in the expression of CCR6^+^IL-17A^+^ (dual positive) T-cells was observed in the presence of Itolizumab as compared to Iso Ab (Fig 4D).

Itolizumab had little impact on the IL-17 ^negative^ CCR6^+^ (non-Th17) cells, thereby probably sparing resting memory T-cells (Fig 4D). These results suggest that the inhibitory effect of Itolizumab might be restricted to CCR6 positive effector T-cell subset. These experiments prove that Itolizumab inhibits the activation and differentiation to Th17 cells by inhibiting key transcription factors pSTAT3, RORγT and hence results in reduced frequency of IL17 secreting Th17 effector cells (reduced RORγT^+^ IL-17A^+^ and CCR6^+^IL-17^+^ T-cells effector subsets).

Phenotype of PBMCs that were left unstimulated in culture from day 3 till day 13 is shown in S6 Fig. This confirms that undifferentiated PBMC were mainly secreting IFN-γ when given a short stimulation with PMA ionomycin.

The FSc-SSc scatter gating used in all flowcytometry experimental analysis was able to exclude dead cells as shown by representative 7-AAD staining on day3 and day 6 on unstimulated and stimulated cells (S7 Fig).

### Transcriptional alteration over time by Itolizumab in Th17 polarizing conditions

**Fig 5:**
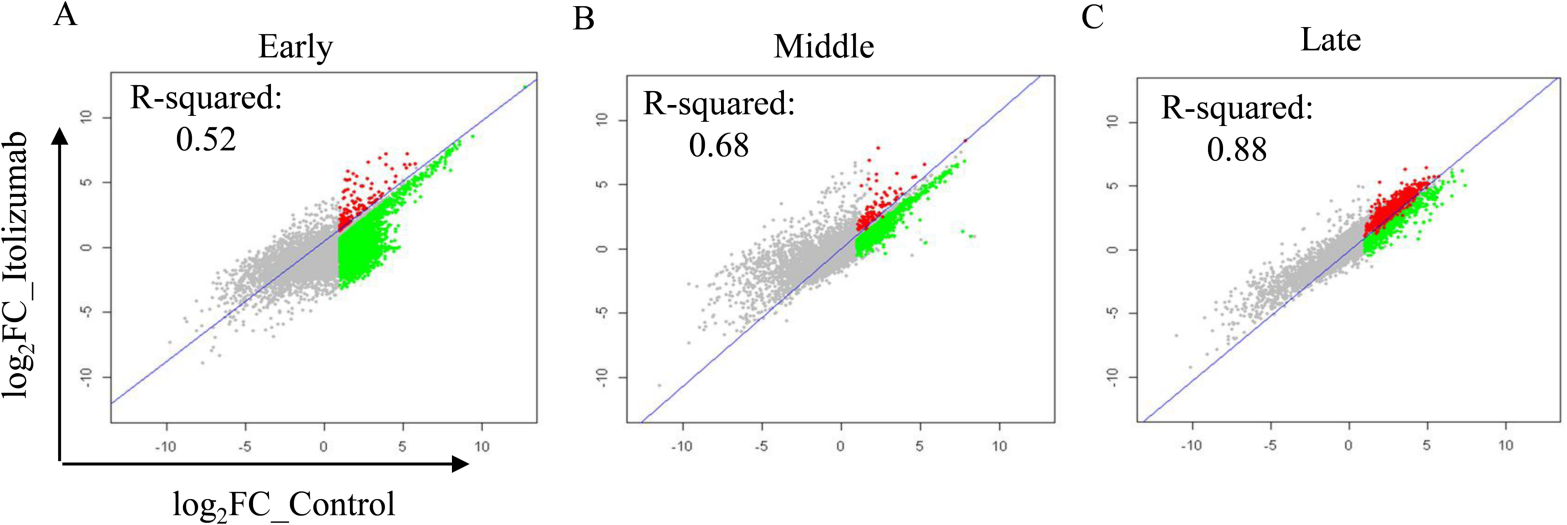
Differential expression profiles over time for Itolizumab. Scatter plots for all the 15K genes in the array for control group versus Itolizumab in early, middle (Average expression for days 6 and 8) or late phases (Average expression for days 10 and 14) are shown. Regression line represents R-Squared value between Itolizumab and Iso Ab groups. The colored region is > log_2_ 1 over unstimulated in the control group. Green region depicts the genes down regulated by at least 20% in the Itolizumab arm over the Iso Ab group.

The effect of anti-CD6 mAb on Th17pol cells relative to Iso Ab treated cells was evaluated in time course studies with analysis on days 3, 6, 8, 10 and 14. Based on the expression pattern of key cytokines evaluated (Fig 3), day 3 was considered “early”, the profile obtained on days 6 and 8 as “middle”, and on days 10 and 14 as “late”. Itolizumab treatment had significant effects on the early transcripts as determined by the low R^-^Squared values as compared to gene expression profile from Iso Ab treated group (Fig 5A). In the middle and late stages, the R^-^Squared values were higher suggesting similarity with control group gene transcription profile (Fig 5B and 5C). This suggests that maximum transcriptional activity is observed early during T cell activation and differentiation and that a larger proportion of genes are transcriptionally altered “early” during T cell activation and differentiation, by anti-CD6 mAb treated cells as compared to Iso ab.

Eighty six percent of genes showing enhanced expression in the control group, (>log_2_1 relative to unstimulated cells) were down regulated by at least 20% in the presence of Itolizumab in the “early” phase. Further, 72.9 and 23.0% of the genes were down regulated by Itolizumab in “middle” and “late” phases respectively (Fig 5, Table 1).

**Table 1:** Differential expression of genes in presence of Itolizumab at different phases relative to control (gene expression custom 15K).

Stages	Early	Middle	Late
No. of probes/genes with Fold Change > = 2 in Control	4795	2233	4118
No. of probes/gene with Fold Change > = 2 in Control and % change > 20 in presence of Itolizumab	4121	1628	950
% genes downregulated with Itolizumab treatment	85.94	72.91	23.07

Percent of genes affected by Itolizumab relative to genes overexpressed in control at three different time points under Th17 polarizing conditions.

**Fig 6:**
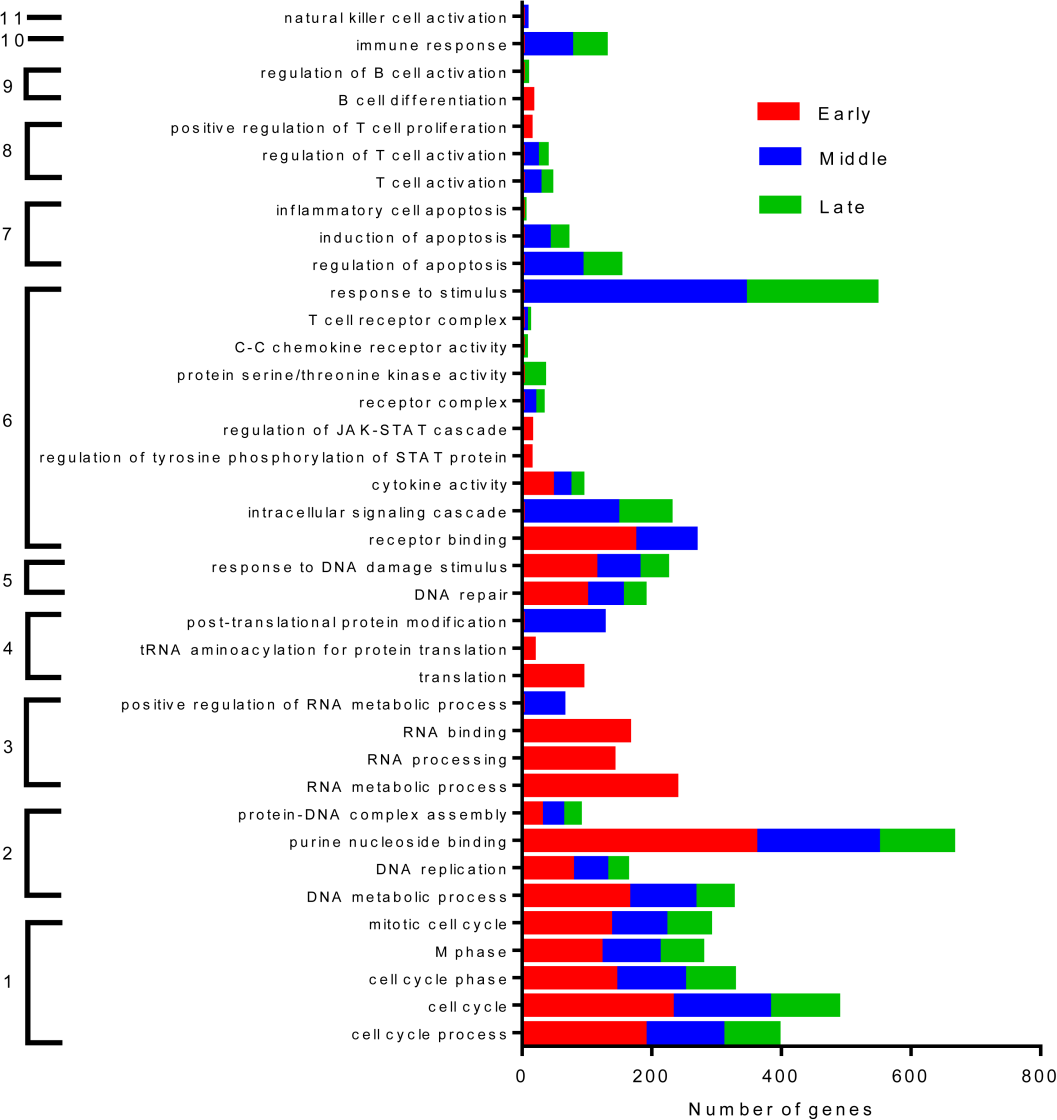
Key functions inhibited by Itolizumab. There are 11 major functional groups significantly impacted by Itolizumab over the early, middle and late phases relative to the Iso Ab control group. They are 1- Cell cycle 2. DNA Metabolism 3. RNA processing 4. Translation 5. DNA damage 6. Signaling 7. Apoptosis 8. T-cell activation 9. B cell differentiation 10. Immune response and 11. NK cell regulation. All the genes listed in the function is a measure of how likely it was that the genes from the data set participate in that function. All functions listed here were highly significant *p≤0.05 using a right tailed Fisher’s exact test.

To understand the broad functional implications on the gene profiling, a clustering of genes based on functional significance was identified. The key functions significantly and consistently altered across early, middle and late phases were genes linked to cell cycle, DNA metabolism, replication and repair. Within the PBMC population, genes inducing positive regulation of T, B cell and other immune cells were significantly inhibited by Itolizumab during certain phases of the experimental conditions (Fig 6).

Functions specifically impacted in the early stage are RNA transcription and translation related genes. In line with the immunomodulatory nature of Itolizumab, apoptosis associated genes are downregulated by Itolizumab thereby reinforcing evidence that, apoptosis or T-cell depletion is not a relevant mechanism of action of this anti-CD6 mAb. While signal transduction pathway like the JAK-STAT is significantly suppressed by Itolizumab treatment during early stage, intracellular signal transduction and chemokine receptor interaction are primarily affected in the middle and the late stages. Based on these functions and from our previous studies [33], we identified a set of 55 genes which were affected in each of the three phases of T cell activation and differentiation under Th17 pol conditions. (S2 Table). 13 genes from this list (IFN-γ, FAS, IL2RB2, LAT, BRCA1, CCR4, CDC2, CDC20, IL26, CD28, IRF4, PLK1, IL-2) were consistently down regulated by Itolizumab at all the phases. These results suggest that Itolizumab affects lymphocyte activation in PBMCs relative to Iso Ab.

### Itolizumab but not its F(ab’)2 fragment inhibits T cell signaling, activation and proliferation

To understand the mechanism for Itolizumab-mediated T cell inhibition, we investigated the role of Itolizumab in inhibiting an activating CD6-ALCAM interaction. While CD6- ALCAM is a well characterized receptor–ligand interaction, some publications have described the presence of an additional ligand binding to domain one of CD6 [55, 56]. To address this issue, we generated the F(ab’)2 fragment of Itolizumab. The hypothesis tested is CD6- ligand interaction is disrupted by both Itolizumab and its F(ab’)2 fragment leading to similar inhibition of T cell activation.

**Fig 7:**
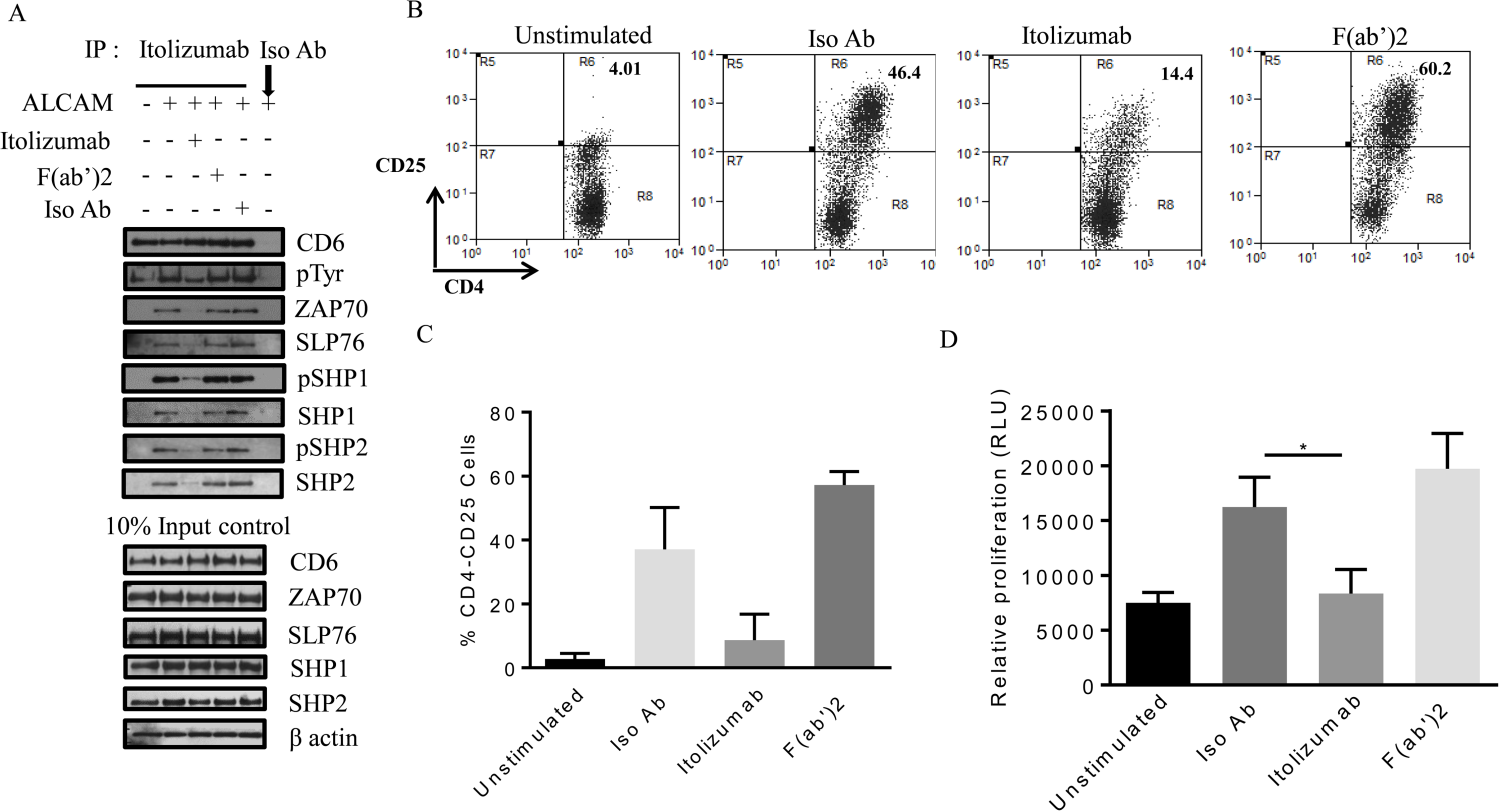
Itolizumab but not its F(ab’)2 fragment inhibits T cell signaling, activation and proliferation. (A) Human PBMCs were plated on ALCAM (10 μg/ml) coated plates for 40 minutes with or without Itolizumab (10 μg/ml), F(ab’)2 fragment of Itolizumab (equimolar amount), or Iso Ab (10 μg/ml). CD6 was immune precipitated with either Itolizumab or Iso Ab and immune blotted for CD6, p-Tyr, Zap70, SLP76, phospho and total SHP1 and SHP2. Corresponding 10% input samples were run as negative controls. Representative blots from at least three independent experiments is shown here. (B-D) Human PBMCs were stimulated with 0.5 ng/ml of anti-CD3 antibody (OKT3), and treated with Itolizumab / Isotype antibody (10 μg/ml), or F(ab’)2 at (equimolar amount) respectively, for 72 h. (B-C) CD25 was used as T cell activation marker in CD4^+^-gated cells using flow cytometer. B shows representative dot plot, while C is the quantification from 2 independent experiments. (D) T cell proliferation was estimated by cell titre glo reagent, and bar graph shows mean ± SD values from 2 independent experiments each done in triplicate *p≤0.05. For Fig 7D non-parametric one way ANOVA statistical analysis was used using Dunn’s multiple comparison analysis.

To evaluate signal transduction downstream to CD6, human PBMCs were stimulated with plate bound ALCAM [1] in presence or absence of Itolizumab or its F(ab’)2 fragment. Using this technique, ALCAM dependent CD6 phosphorylation and CD6-interacting molecules from immune-precipitated CD6 protein was investigated (Fig 7). Equal CD6 pull down by Itolizumab was confirmed by CD6 immuno blot using 2 different antibodies Itolizumab and MEM-98 (Figs 7A and S9A). We investigated Tyr phosphorylation of pulled down CD6 protein, and found out that ALCAM-CD6 interaction increased CD6 tyrosine phosphorylation by over 2.5 fold. This increase in phosphorylation was inhibited by Itolizumab but not by its F(ab’)2 fragment (Figs 7A and S9B). Zap70 (a Kinase) and SLP76 (a docking protein), known binding partners of CD6 [2, 57] were examined for their association with immunoprecipitated CD6. ALCAM-CD6 interaction increased SLP76 and Zap70 association with CD6 by 3–4 fold, and again this was inhibited by Itolizumab but not by its F(ab’)2 fragment (Figs 7A, S9C and S9D). Phosphorylation of receptors is controlled by expression of phosphatases. SHP1 and SHP2 are key phosphatases known to be associated with receptor proteins and control their phosphorylation thereby modulating signal transduction [58, 59]. We evaluated the association and phosphorylation of these proteins with immunoprecipitated CD6 to understand the role of these phosphatases in Itolizumab-mediated inhibition. As shown in Figs 7A, S9E and S9F, ALCAM-CD6 interaction increased phosphorylation of CD6 associated SHP1 and SHP2 by 3–4 fold. In addition, Itolizumab but not its F(ab’)2 fragment inhibited both total and phosphorylated (activated) SHP1 and SHP2 associated with CD6 thereby bringing their expression to baseline levels (Figs 7A, S9E and S9F).

To understand the significance of these signal transduction events on the activation and proliferation of T cells, human PBMCs were stimulated with soluble anti CD3 antibody (OKT3 clone) in presence of Itolizumab, or F(ab’)2 fragment or Iso Ab for 72h. As shown in Fig 7B–7D, Itolizumab but not it’s F(ab’)2 fragment inhibited CD25 activation marker in CD4^+^gated cells and T cell proliferation by 7 and 2 fold respectively. Overall these results indicate that a key mechanism of action of Itolizumab involves a decrease in an activating ALCAM-CD6 co stimulatory signal by directly reducing CD6 hyper phosphorylation and preventing the docking of key molecules associated with T cell signaling, activation and proliferation. Since, F(ab’)2 fragment of Itolizumab fails to exert this effect we conclude that Itolizumab does not inhibit the putative ligand CD6 D1 interaction but disrupts an activating ALCAM-CD6 D3 classical interaction.

### Mouse anti CD6 domain 1 antibody (m CD6D1 mAb) ameliorates EAE in mice

**Fig 8:**
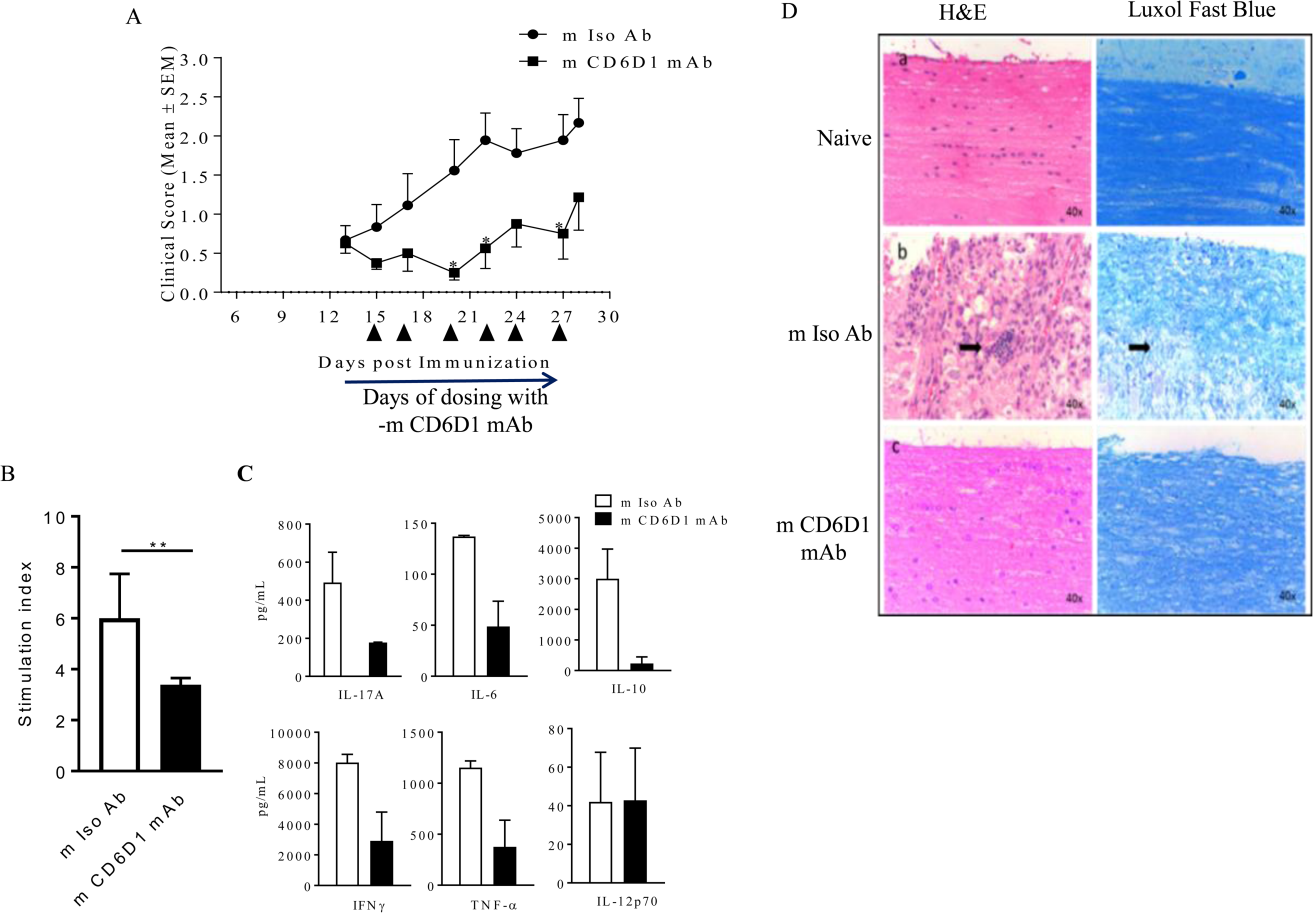
Mouse anti CD6 domain 1 antibody (m CD6D1 mAb) ameliorates EAE in mice. EAE disease was induced in C57BL/6 normal female mice using MOG_35-55_ emulsified in CFA and pertussis toxin. Randomized mice were injected with 60 μg in 100 μl volume of either control m Iso Ab (shown in circles) or m CD6D1 mAb (shown as squares) between day 15 and day 27 (arrows indicate days of dosing). 7 animals were used in each group. (A) Clinical scores are represented as Mean ± SEM. Fig is representative of six independent studies (*p≤0.05). The statistical significance was determined using 2 way ANOVA followed by Sidak’s multiple comparison analysis. (B-C) Splenocytes of these mice were stimulated with plate bound anti-mouse CD3 mAb. (B) After 96 h, proliferation of these splenocytes was estimated using Alamar Blue reagent **p≤0.01. Statistical significance was determined using non-parametric unpaired t-test followed by Mann-Whitney test (C) Supernatants were collected after 72 h and 6 cytokines were analyzed using Cytokine bead array (CBA) kit. All cytokines except IL-12 showed significant difference using unpaired t-test (*p≤0.05). (D) m Iso Ab and m CD6D1 mAb treated EAE-induced mice were sacrificed on day 32 post immunization and transverse lumbar spinal cord sections were prepared (middle and lower panel). Naive mouse without EAE induction was used as additional control (top panel). Left panel showed light microscopy of sections stained with hematoxylin and eosin (H&E), while the right panel showed luxol fast blue staining for demyelination of neurons. Arrows in the middle panel show infiltrating lymphocytes among neutrophils migrating into the spinal cord in mice with disease and treated with m Iso Ab. Representative Fig shown.

To understand the significance of our cell culture results, we investigated the effect of non-depleting anti- mouse CD6 domain 1 antibody (m CD6D1 mAb) in Experimental autoimmune encephalomyelitis (EAE) disease mouse model of MS. Characterization of this antibody is shown in S11 Fig and S3 Table. As shown in Figs 8A and S12A, EAE-induced animals treated with m CD6D1 mAb showed decreased clinical score in comparison to m Iso Ab (the average maximum reduction over control in EAE score during dosing duration for 6 independent studies was 62% ± 24% with a range of 44%–100% (S4 Table).

At the end of the study, splenocytes harvested from mice treated with m Iso Ab or m CD6D1 mAb were stimulated *ex vivo* with anti-mouse CD3 antibody as well as more physiologically relevant MOG specific antigen. In both cases, m CD6D1 mAb showed a hypo-proliferation compared to m Iso Ab by almost 1.5 fold (Figs 8B and S12B). IL-17A, IL-6, IL-10, IFN-γ and TNF-α cytokines analysis in cell supernatants from anti CD3-mediated stimulation showed 64%, 64%, 92%, 64%, 67% inhibition while MOG-mediated *ex vivo* stimulation showed 91%, 91%, 57%, 79%, 11% inhibition respectively for m CD6D1 mAb compared to m Iso Ab treated animals (Figs 8C and S12C). Therefore, in line with the observations of immunomodulation by Itolizumab by suppressing pro inflammatory cytokines in human cells, m CD6D1 mAb shows comparable immunomodulatory effects in mouse *in vivo* models.

To understand the effect of demyelination with this m CD6D1 mAb histopathological analysis (H&E and Luxol fast blue staining) of the spinal cord transverse lumbar sections was done. Luxol fast blue staining showed reduced demyelination in mice treated with m CD6D1 mAb suggesting neuroprotection as compared to the control antibody treated mice (Fig 8D). This neuroprotection observed in the m CD6D1 mAb treated animals was associated with reduced infiltration of lymphocytes and polymorphonuclear cells in the spinal cord (shown by arrows in middle panel for m Iso Ab treated group). Fifty seven percent (4/7 animals) showed inflammation and demyelination in control group, whereas none (0/7 animals) showed any inflammation and demyelination (representative figure shown in Fig 8D). Taken together these results show the effect of m CD6D1 mAb in EAE disease mouse model of MS and re-iterates the importance of Itolizumab as a potential therapeutic target in autoimmune diseases such as multiple sclerosis.

## Discussion

T-cell activation, differentiation and function is controlled by co-stimulatory and co-inhibitory receptors with diverse expression, structure and function, and is largely context dependent. Though some of the best characterized co-stimulatory molecules such as CD28 and co-inhibitory receptor for example, CTLA4 are very well studied [60], until recently, little was known about CD6 lymphocyte receptor. Although, the exact signal transduction pathway via CD6 has not been clearly elucidated, recent articles show an important role of CD6 in T-cell signaling. Roncagalli et al., show that activation of TCR and subsequent phosphorylation of ZAP70 facilitated CD6 association to the TCR complex. Here, CD6 acts like a scaffold protein permitting the recruitment of SLP-76 and the guanine nucleotide factor Vav1 independent of LAT, an adaptor or docking protein [2]. In addition, hyper phosphorylation at tyrosine, serine and threonine residues on the cytoplasmic tail of CD6, leads to CD6 binding to adaptor molecules such as SLP76 followed by time and dose dependent MAPK activation [1, 9, 33, 57, 61]. Unlike CD5, another member of the scavenger receptor superfamily and having close homology to CD6, clearly identified as a co-inhibitory molecule, the role of CD6 in T cell modulation is still controversial [38, 61–63]. Though there are significant number of articles supporting the co-stimulatory role of CD6, there are some publications which position CD6 as a co-inhibitory molecule. In an earlier study, CD6 was identified as a signaling attenuator whose expression alone, i.e. even in the absence of ligand engagement was sufficient to restrain signaling in T-cells [37]. Further recently, Mascaro et al., have shown that in CD6 null mice there is a negative selection in thymus and an increased activation in response to self- or environmental antigens in the periphery. This finding is indicated by an expansion of T cell subsets with memory and regulatory phenotypes, indicating an inhibitory function for CD6 [64]. In addition, there are couple of recent articles that show dual role of CD6 and advocate CD6 as both positive and negative regulator of T cell [7, 56]. The controversy continues even for expression level of CD6 receptor. In autoimmune conditions such as multiple sclerosis, while one study showed lower expression of CD6 mRNA in patients than in healthy individuals [65], a recent report suggests higher CD6 expression in peripheral blood T-cells as well as cerebrospinal fluid (CSF) T-cells in patients with active lesions [66]. Another article reports about clones of Th17 cells derived from CSF of MS patients that show increased CD6 expression [19].

In this study, using human PBMCs, we show that anti-CD3 and anti-CD28 induced activation of T cells results in increased expression of CD6 receptors from baseline. Further, we report that in Th17 pol conditions the median expression and absolute receptor counts for CD6 on T-cells are increased even further than when stimulated in Thnp condition (Fig 1D and 1E). While the increased expression of CD6 under PMA stimulation [67] and the increased mRNA expression in PHA stimulated PBMC [68] has been reported, to our knowledge this is the first time that the increased expression of CD6 receptor *in vitro* under a TCR mediated stimulation along with co-stimulation has been quantified.

CD6 antibodies which recognize CD6 domain 1, not involved in ligand binding, have been shown to inhibit CD6/ALCAM interactions between cells [69]. Anti-CD6mAb (M-T605) that binds to SRCR domain 1 of CD6, CD6-ALCAM blocking antibodies such as anti-ALCAM (3A6, anti-CD166) and anti-CD6-D3 (OX126) have reported contrasting functions. While anti ALCAM antibody enhanced T-cell proliferation interestingly, anti-CD6D1 and D3 antibodies inhibited the same [37, 70].

Itolizumab binding to domain 1 of CD6 independent of its ligand (ALCAM) binding has had considerable success in recent clinical trials [29, 31]. We show here that even under strong “classical” co-stimulation, with anti-CD3 and anti-CD28, Itolizumab significantly reduced activation (decrease in CD25), proliferation and differentiation to effector T-cells. These results indicate that in these mixed population of PBMCs in proliferation assays, a sustained interaction with enhanced CD6 and increased ALCAM on cells is required for the continued activation and proliferation process of T-cells.

In all our *in vitro* experiments, we have consciously used physiologically equivalent concentration of Itolizumab. The clinically relevant dose of Itolizumab ranges from 0.4–1.6mg/kg which is equivalent to 10–40 μg/ml dose *in vitro*. Receptor occupancy studies indicate that CD6 receptors are fully saturated at this range of Itolizumab. In addition, in our previous paper we have shown that the maximum inhibitory potential of Itolizumab also lies within this range [33].

Pathogenic Th17 cells play an important role in autoimmune disorders such as multiple sclerosis and psoriasis. In regular, human T cell activation in non-polarizing condition, the percentage of activated Th17 cells is very low and therefore to study the impact of the drug on Th17 subset is difficult [71, 72].

Here we successfully establish Th17 lineage (Fig 1A) and used this tool to evaluate the effect of Itolizumab in these differentiating T effector cells. Our results indicate that Itolizumab inhibits T cell activation and differentiation to effector Th17 cells. Key proteins such as IFN-γ, TNF, IL-2, IL-2R, STAT3, IL-17 were found to be down regulated with Itolizumab both in the array as well at the protein level as shown in Figs 3, 4 and 6 and S2 Table. These results position Itolizumab as an upstream modulator by primarily inhibiting the activation and differentiation of T-cells into effector T-cells. The effect is achieved not only in standard proliferating conditions but also during Th17pol conditions by affecting TCR mediated signal transduction and suppression of key effector cytokines such as IFN-γ, IL-17 and IL-2.

**Fig 9:**
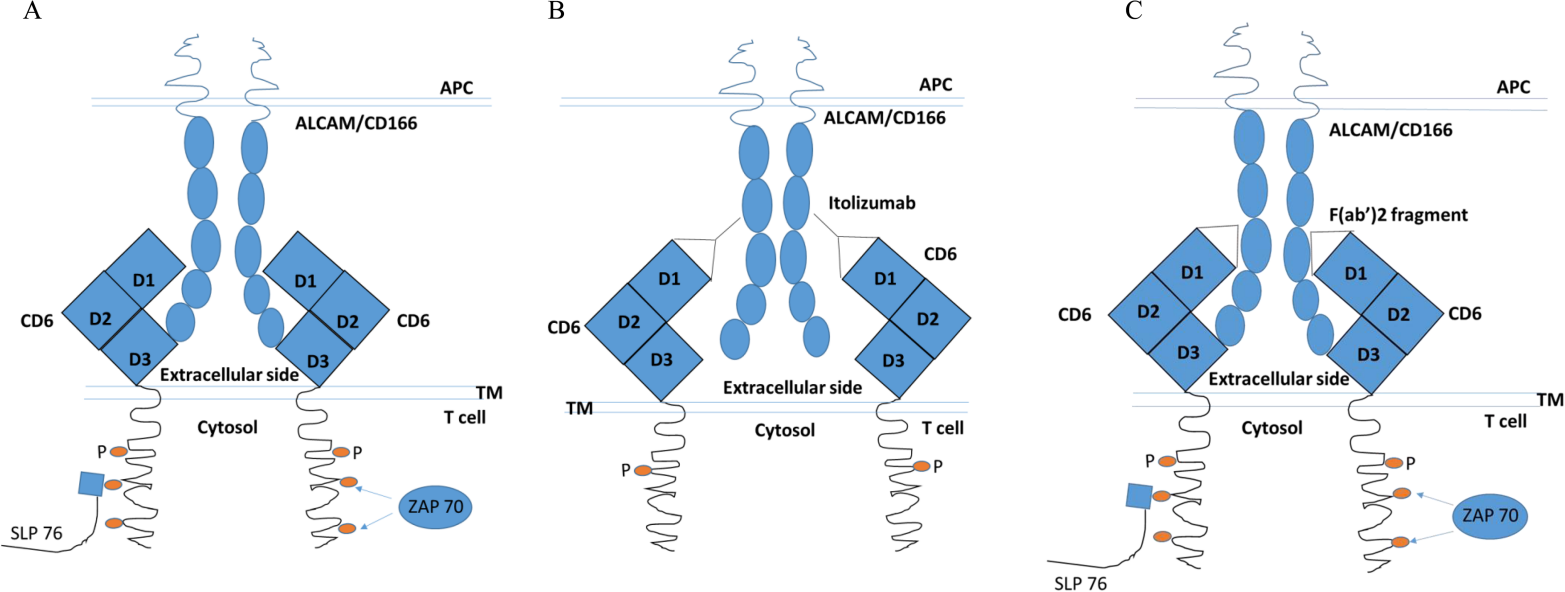
Cartoon depicting the proposed mechanism of action of ALCAM-CD6 interaction. A. ALCAM–CD6 interaction in the absence of any antibody. B. ALCAM-CD6 signal is blocked by Itolizumab. ALCAM–CD6 interaction is not affected by Itolizumab F(ab’)2 fragment.

Here, in our model of ALCAM-CD6 interaction (Fig 7), that closely resembles the CD6 clustering at immune synapse [37], we show enhanced CD6 receptor phosphorylation which is associated with docking of key signaling molecules such as Zap70 and SLP76. Receptor phosphorylation is controlled by the association of receptors with phosphatases which then modulate signal transduction events mediated by ligand receptor interaction [58, 59]. Among the well-studied phosphatases are SHP1 and SHP2 [73–76]. The functions of these phosphatases are not completely understood but literature suggests that while SHP1 blocks receptor activation, SHP2 is associated with receptor stimulation [73, 74]. However, in the presence of Itolizumab, both total SHP1 and SHP2 as well as their phosphorylated entities show substantially reduced association with CD6 (Fig 7). Itolizumab therefore does not mediate its T cell inhibitory effect by enhancing the association of SHP1 or pSHP1, a well-known inhibitory phosphatase [74] with the CD6 receptor complex. The fact that Itolizumab can prevent a CD6 activating signal mediated by ALCAM is further confirmed with a more physiological activation of T cells with anti-CD3 antibody (S12 Fig). In our experiments, F(ab’)2 fragment of Itolizumab does not prevent T cell signaling, activation and proliferation. Therefore, our results are in agreement with previous studies [35, 47] which show that at the molecular level, CD6 domain one targeting antibodies prevent the optimal engagement of CD6-ALCAM critical for T-cell activation. We propose a model for this interaction between ALCAM-CD6 in the presence of Itolizumab in Fig 9. Accumulation (clustering) of CD6 at the immunological synapse [38] initiates activation of CD6 receptor by interacting with ALCAM on antigen presenting cells. In this model we suggest that Itolizumab upon binding to domain one of CD6 provides a steric hindrance and prevents the optimal interaction of ALCAM with CD6 D3. This results in the attenuation of T cell signaling mediated by this costimulatory molecule CD6. Under other circumstances, where CD6 is not clustered, Itolizumab does not prevent or interfere with ALCAM- CD6 interaction as was reported earlier [28, 33]. At the immunological synapse membrane distances between APCs and T cells are around 15 nm [11]. Extrapolation of the five ECD of ALCAM (CD166) (two IgSF V and three C domains) is ~ 20 nm while CD6 nonlinear structure of three SRCR domain spans ~7.8 nm [47]. This would indicate that at the synapse a significant bending of the receptor–ligand pair of ALCAM-CD6 has to take place. It is reasonable to estimate that a full length IgG1 antibody (Itolizumab) of around 10 nm can disrupt adequate receptor–ligand interaction for optimal signaling but a smaller fragment of the antibody F(ab’)2 at around 3-4nm would fail to inhibit this interaction. Further, the failure of F(ab’)2 fragment to prevent CD6 activation and subsequent T cell proliferation would indicate that the primary mode of action of Itolizumab is to inhibit an activating ALCAM-CD6 interaction as shown in Fig 9.

The preclinical *in vivo* study in this report further validates the *in vitro* findings. Here we report for the first time the successful use of a m CD6D1 mAb in ameliorating EAE in a therapeutic setting. The binding site of Itolizumab to human SRCR1 of CD6 is not identified. Alonso R et al have reported putative epitopes from a random phage library against T1 specific phagotopes [28]. They show that UMCD6 an anti CD6 mAb and Itolizumab bind to a specific consensus motif RXE/Q. Recently, Chappell et al have solved the 3D structure of human CD6 ECD [47]. To identify the epitopes specific to different anti CD6 antibodies towards SRCR1, they made alanine mutants on number of surface-exposed residues. They identified point mutations at E63A and R77A (S13B Fig) which affects binding to MEM98 and MT605 anti CD6 mAbs, respectively. Interestingly, MEM98 is known to compete with Itolizumab as shown in S3 Fig. MT605 partially competes with Itolizumab [77] and this is clear from the distinct regions to where they probably bind as shown in S13B Fig. Like MEM98, UMCD6 is also known to compete with Itolizumab binding [28]. However, independent studies suggest that irrespective of different epitopes mapped to SRCR1 of CD6, both MT605 and Itolizumab inhibit T cell activation suggesting that binding to SRCR1 domain is more critical than an exact binding location. No anti-mouse SRCR1 specific antibody has been reported so far and we are the first to report functional inhibition with a murine specific antibody. The sequence alignment of SRCR1 domain ([28] S13A Fig) of CD6 indicates only 54% identity between human and mouse which in turn suggests a potentially new binding region for the murine specific antibody. Further, it demonstrates that the RXE/Q consensus motif and R77 is not conserved in the mouse CD6 SRCR1 domain. However, E63 position of mouse SRCR1 domain is still present in the epitope 1 region. Furthermore, to visualize the epitope 1 and 2 regions, mouse SRCR1 domain was modelled using the human CD6 as template (PDB: 5A2E) to demonstrate different sequences in the mAb binding region (S13B Fig). The exact epitope of binding of Itolizumab to human CD61 and of this murine antibody to SRCR1 of mouse CD6 needs to be further investigated. Determination of the X-ray crystal structures of mAb antibodies (Itolizumab, UMCD6, MT605 etc.) bound to CD6 SRCR1 domain (human and mouse) antibodies will allow us to map more accurate model of how these proteins regulate interactions at the cell surface, and to design the most effectively CD6 mediated antibody therapy.

Overall, together with substantial clinical evidence we position Itolizumab as an immunomodulator required to disrupt an activating CD6-ALCAM interaction.

### Post script

While this paper was under review, a very significant paper validating the role of CD6 in autoimmune diseases specifically multiple sclerosis has appeared. Li et al [56] very elegantly demonstrated that in CD6 KO mice EAE is suppressed. They also generated a humanized CD6 expressing mice and used UMCD6 antibody [antibody known to compete with Itolizumab for CD6 binding [28] to study the effect of the drug in the EAE model. Identical to our studies reported here with a mouse SRCR1 CD6 mouse antibody, this human SRCR1 specific CD6 antibody was not depleting and suppressed EAE. Intriguingly in the CD6KO mice AICD was observed and not with the antibody treated animals. The authors have speculated on the presence of a domain one specific ligand to explain the EAE amelioration phenotype. However, our results with F(ab’)2 support disruption of ALCAM-CD6 interaction rather than domain 1 specific ligand mediated CD6 activation.

Recently Lécuyer MA [78] reported that in ALCAM KO mice EAE was exacerbated but this was associated with loss of integrity in Blood Brain Barrier (BBB) endothelial cells because of lack of ALCAM allowing the migration of T cells into the central nervous system and causing inflammation there.

## Supporting information

S1 Fig Increased expression of CD6 on activated T cells in Th17 polarizing conditions. PBMCs were left unstimulated (shaded histogram) or stimulated in Thnp or Th17pol conditions. CD6 expression (using biotinylated Itolizumab as detection reagent) was analyzed on Day 9 and plotted as CD6 overlay histograms gated on lymphocyte scatter and CD4^+^ T cells. Data is representative of 6 different donors.
(DOCX)

S2 Fig Increased CD6 expression on CD8^+^T-cells. Receptor density of CD6 expression in unstimulated, Thnp and Th17pol conditions on gated CD8^+^ lymphocytes is shown as a scatter plot using biotinylated Itolizumab as detection reagent. An increasing trend in CD6 receptor expression is seen in Thnp and Th17 pol conditions over unstimulated cells. Data represents 3 donors.
(DOCX)

S3 Fig CD6 receptor on lymphocytes is not internalised and is occupied with Itolizumab. Human PBMCs were left unstimulated or stimulated with soluble anti CD3 0.5 ng/ml (OKT-3) in presence of Iso Ab or Itolizumab at 10 μg/mL for 3 days. Post stimulation, cells were harvested and stained with anti CD6 Ab, MEM98 clone (A) and anti-human IgG, Fc specific (B). In panel A, since the CD6 receptor is occupied with Itolizumab, MEM 98 (commercially available anti CD6 D1) could not bind in Itolizumab treated groups and hence no signal is observed. The positive signal with anti-human IgG, in Itolizumab treated group in panel B suggests that Itolizumab is occupying CD6 receptor on lymphocyte surface. Data is representative of at least 3 independent experiments.
(DOCX)

S4 Fig Itolizumab inhibits IFN-γ and IL17-A expression in CD8+T cells. Human PBMCs were stimulated with anti-CD3 and anti-CD28 beads or soluble anti CD3 0.1 ng/ml (OKT3) and sol anti CD28 (10 ng/ml) in Th17pol conditions in presence of Itolizumab or Iso Ab at 40 μg/mL. On day 6, cells were re-stimulated with PMA-Ionomycin for 5 hours and analyzed for expression of intracellular cytokine IFN-γ and IL-17A. Representative flow cytometry dot plots (gated on lymphocyte scatter and CD8+ lymphocytes) on day 6 are shown in Fig. Percent cells are indicated in the quadrants Itolizumab substantially inhibits IFN-γ and IL-17A expression in CD8+ lymphocytes. Data is representative of 2 independent experiments.
(DOCX)

S5 Fig Itolizumab does not induce AICD in stimulated PBMC. (A) Human PBMCs were left unstimulated or stimulated with soluble anti CD3 0.5 ng/ml (OKT-3) in the presence of Iso Ab or Itolizumab at 10 μg/mL for 3 days. Post incubation, cells were harvested and stained with anti CD3, Annexin V and 7-AAD. The % Annexin V positive, 7-AAD negative CD3^+^T cells has been plotted. The bar graphs show mean±SD from 3 independent experiments. (B) Similar experiment as described in panel A with staining at different time points was done to analyse AICD across days. At each time point, cells were harvested and stained with CD3, Annexin V and 7-AAD. The % Annexin positive, 7-AAD negative CD3^+^ T cells has been plotted. Data is from one experiment.
(DOCX)

S6 Fig Phenotyping of unstimulated human PBMCs using IL-17 and IFN-γ intracellular cytokine expression across days. (A) PBMCs were left unstimulated for 3 days and analysed for expression of intracellular cytokine IFN-γ and IL-17A. Representative flow cytometry dot plots (gated on lymphocyte scatter and CD3^+^ T-cells) is shown. Percent T-cells are indicated in the quadrants. (B) PBMCs were left unstimulated for 3, 6, 8 and 13 days. Cells were re-stimulated with PMA-Ionomycin for 5 hours and analyzed for expression of intracellular cytokine IFN-γ and IL-17A. Representative flow cytometry dot plots (gated on lymphocyte scatter and CD3^+^ T cells) across days are shown. Percent T-cells are indicated in the quadrants. In the panels, before gating on lymphocyte gate, total cells were selected and gated to get uniform event count display.
(DOCX)

S7 Fig Lymphocyte gate based on SSC and FSC excludes 7AAD positive (dead) cells. PBMCs were left unstimulated or stimulated with soluble anti CD3 0.5 ng/ml (OKT-3) for 3 days / 6 days. Post incubation, cells were harvested and stained with 7-AAD. Panel A shows the representative lymphocyte gate that is put in all experiments. The dead cells (7-AAD positive) seen in green are excluded out of the gate. In panel B, no gate has been applied and positive signal is seen with 7-AAD, indicating the presence of dead cells. In panel C, lymphocyte gate has been applied and the cells do not stain positive for 7-AAD, indicating healthy cell population. Day3 7-AAD staining is a representative of 3 independent experiments and Day6 7-AAD staining is from a single experiment.
(DOCX)

S8 Fig Coomassie Blue staining for F(ab’)2 fragment of Itolizumab. (A) Undigested Itolizumab (1) and F(ab’)2 fragment of Itolizumab (2) run in non-reducing gel. For undigested Itolizumab band was seen around 150 kDa, while in F(ab’)2 fragment band was seen around 100 kDa. (B) Undigested Itolizumab (2) and F(ab’)2 fragment of Itolizumab (3) run in reducing gel. For undigested Itolizumab two bands were seen around 25 and 50 kDa respectively, while in F(ab’)2 fragment only one band was seen around 25 kDa.
(DOCX)

S9 Fig Itolizumab but not its F(ab’)2 fragment inhibits T cell signaling, activation and proliferation. (A) CD6 Western blot of CD6 immune precipitated samples using MEM-98 antibody. (B-F). Quantification (mean±SD) of p-Tyr, Zap70, SLP76, p-SHP1 and p-SHP2 relative intensity as shown in Fig 7A of the manuscript. (B-D) showed a significance difference (p≤ 0.05) while (E-F) showed a trend for reduction with p values 0.1 and 0.2 respectively, when compared between Iso Ab and Itolizumab.
(DOCX)

S10 Fig Characterization of m CD6D1 mAb. (A) Mouse anti CD6 monoclonal antibody (m CD6D1 mAb) binds to Domain 1 of mouse CD6: Anti CD6 mAb was screened for its binding to plate coated full length, domain-1 and domain-2 of mCD6-Fc by ELISA. mALCAM, hCD6-Fc and anti hCD6 MAb (Itolizumab) were used as controls. Representative graph is shown with mean±SD of two independent experiments. (B) Inhibition of proliferation: Naive mouse splenocytes were added to plate coated with anti CD3 mouse Ab in presence or absence of different concentration of soluble m CD6D1 mAb (2.5–40 μg/ml). Percent Inhibition of proliferation of these splenocytes was calculated with respect to isotype control using Alamar Blue fluorescent dye. Representative graph from three independent experiments is shown. Data is expressed as mean ± SEM. Range of inhibition of m CD6D1 mAb to activated splenocytes is similar to that of Itolizumab on activated T cells. (C) ALCAM binding is independent of m CD6D1 mAb binding to CD6: Mouse CD6-Fc chimera coated plates were incubated with m CD6D1 mAb and/or increasing dose of mouse ALCAM-Fc chimera and binding of m CD6D1 mAb to CD6 was examined. m Iso Ab has a very minimal binding to plate coated mouse CD6-Fc.
(DOCX)

S11 Fig m CD6D1 mAb treated EAE-induced mice have hypo-proliferation and lower cytokine release with *ex vivo* MOG treatment. EAE disease was induced in C57BL/6 normal female mice using MOG_35-55_ emulsified in CFA and pertussis toxin. Randomized mice were injected with 60 μg in 100 μl volume of either control m Iso Ab (shown in circles) or m CD6D1 mAb (shown as squares) between day 15 and day 27 (arrows indicate days of dosing). 8 animals were used in each group. (A) Clinical scores are represented as Mean ± SEM. (*p≤0.05). (B-C). Splenocytes of these mice were stimulated with soluble MOG antigen peptide (*p≤0.01). (B) Proliferation of these splenocytes were estimated after 96 h using Alamar Blue reagent (C) Supernatants were collected after 48 h and 6 cytokines were analysed using Cytokine bead array (CBA) kit. All cytokines except IL2 and IL-10 showed significant statistical difference using unpaired t-test (*p≤0.05). For IL-10 the p value is 0.07, while for IL-2 only 1 animal had detectable value.
(DOCX)

S12 Fig Itolizumab inhibits CD6 co-stimulatory signaling in physiologically relevant TCR activation signal. (A) CD6 Western blot of CD6 immune precipitated samples using MEM-98 antibody. (B-F). Quantification (mean±SD) of p-Tyr, Zap70, SLP76, p-SHP1 and p-SHP2 relative intensity as shown in Fig 7A of the manuscript. (B-D) showed a significance difference (p≤ 0.05) while (E-F) showed a trend for reduction with p values 0.1 and 0.2 respectively, when compared between Iso Ab and Itolizumab.
(DOCX)

S13 Fig Sequence alignment and three dimensional structural comparison of Human CD6. (A) Sequence alignment of Human CD6 SRCR1 (Uniprot ID: P30203) and mouse CD6 SRCR1 (Uniprot ID: Q61003) domains was performed using the Clustal omega (http://www.ebi.ac.uk/Tools/msa/clustalo/). The identity and similarity of the residues was found to be 54.5%, and 64.3% respectively with one gap in the alignment. Secondary structure elements derived from the human CD6 extracellular SRCR domain structure is shown above the sequences. Conservative substitutions are shown in red font and identical residues in white font on a red background. The consensus motif RxE/Q epitope for potential binding to therapeutic mAbs is marked on top with a line. The epitopes identified from point mutants study from Chappell et al., work, the E63 and R77 residues are denoted with # and * respectively. The alignments were generated using the ESPript server (http://espript.ibcp.fr/ESPript/ESPript/). (B) The mouse CD6 SRCR1 domain was modelled using the human CD6 SRCR as template (PDB: 5A2E) using the SWISS-MODEL program (https://swissmodel.expasy.org/). The consensus motif RxE/Q predicted for Itolizumab and UMCD6 mAbs binding, E63 (epitope 1) and R77 (epitope 2) epitopes mapped for binding to MEM98 and MT605 mAbs respectively are marked in the human CD6 SRCR1 domain. The same regions are mapped based on the sequence alignment are marked in the mouse CD6 SRCR1 domain. The numbering of the residues are provided based on the published human CD6 structure (5A2E.PDB). The disordered loop region in both the molecules is shown in grey colour. The N and C regions are marked for orientation of the molecule. All the Figs are prepared using the The PyMOL molecular graphics software (http://www.pymol.org).
(DOCX)

S1 Table Increase in CD6 MFI on CD4 and CD8 lymphocytes upon activation. Human PBMCs were left unstimulated or stimulated with anti-CD3, anti-CD28 beads. CD6 expression (using MEM98 as detection reagent) was analyzed on Day 3. The MFI (median fluorescent intensity) of CD6 on unstimulated and stimulated PBMCs gated on CD4^+^ lymphocytes and on CD4^+^CD25^hi^ lymphocytes has been tabulated. Data is shown for 3 independent experiments. Representative gating strategy used for gating on CD4^+^/ CD8^+^ lymphocytes (gate **1**) and CD4^+^CD25^hi^ / CD8^+^ CD25^hi^ lymphocytes (gate **2**).
(DOCX)

S2 Table 55 Genes from the current microarray were selected based on previous studies and literature. (DOCX)

S3 Table Binding kinetics of m CD6D1 mAb, Itolizumab full length and its F(ab’)2 fragment to their respective full length CD6 ligand by surface plasmon resonance method. (DOCX)

S4 Table Percentage reduction in EAE clinical score during treatment over the control in 6 independent EAE studies. (DOCX)

## Abbreviations

Thnp condition: PBMCs stimulated with anti-CD3/anti-CD28 beads
Th17pol condition: PBMCs stimulated with anti-CD3/anti-CD28 beads in Th17 polarizing milieu
MFI: Median Fluorescence Intensity
mAb: monoclonal antibody
m Iso Ab: non specific control antibody for mouse specific antibodies
m CD6D1 mAb: anti mouse CD6 D1 specific monoclonal antibody
Iso Ab: non specific control human antibody (Nimotuzumab)
