# Correction: T cell activation and differentiation is modulated by a CD6 domain 1 antibody Itolizumab

**DOI:** 10.1371/journal.pone.0192335

**Published:** 2018-01-30

**Authors:** Usha Bughani, Arindam Saha, Anshu Kuriakose, Reshmi Nair, Ravindra B. Sadashivarao, Rasika Venkataraman, Swati Patel, Anuja Tushar Deshchougule, Kumar S. Satish, Enrique Montero, Harish V. Pai, Dinesh V. Palanivelu, Ramakrishnan Melarkode, Pradip Nair

S3 Fig is incorrect. In panels A and B, the legend for Iso Ab and Unstimulated have been interchanged. Please view the corrected S3 Fig below.

## Supporting information

S3 Fig CD6 receptor on lymphocytes is not internalised and is occupied with Itolizumab. Human PBMCs were left unstimulated or stimulated with soluble anti CD3 0.5 ng/ml (OKT-3) in presence of Iso Ab or Itolizumab at 10 μg/mL for 3 days. Post stimulation, cells were harvested and stained with anti CD6 Ab, MEM98 clone (A) and anti-human IgG, Fc specific (B). In panel A, since the CD6 receptor is occupied with Itolizumab, MEM 98 (commercially available anti CD6 D1) could not bind in Itolizumab treated groups and hence no signal is observed. The positive signal with anti-human IgG, in Itolizumab treated group in panel B suggests that Itolizumab is occupying CD6 receptor on lymphocyte surface. Data is representative of at least 3 independent experiments.
(DOCX)
