# Supplementary material for: T cell activation and differentiation is modulated by a CD6 domain 1 antibody Itolizumab
Source: PLoS One. 2017 Jul 3;12(7):e0180088. doi: 10.1371/journal.pone.0180088 (PMC5495335; doi:10.1371/journal.pone.0180088)
Supplement: S1 Fig — PBMCs were left unstimulated (shaded histogram) or stimulated in Thnp or Th17pol conditions. CD6 expression (using biotinylated Itolizumab as detection reagent) was analyzed on Day 9 and plotted as CD6 overlay histograms gated on lymphocyte scatter and CD4+ T cells. Data is representative of 6 different donors. (DOCX) [file pone.0180088.s001.docx]

**S1 Fig.**


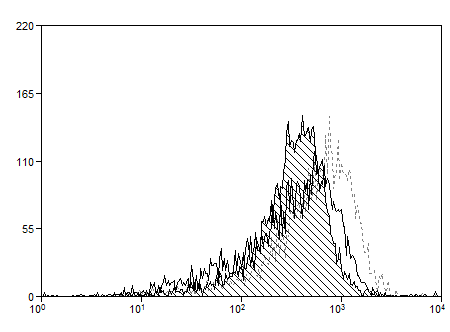


CD6

Th17pol

Thnp

Cell count

Unstimulated

**Increased expression of CD6 on activated T cells in Th17 polarizing conditions**
