# Supplementary material for: T cell activation and differentiation is modulated by a CD6 domain 1 antibody Itolizumab
Source: PLoS One. 2017 Jul 3;12(7):e0180088. doi: 10.1371/journal.pone.0180088 (PMC5495335; doi:10.1371/journal.pone.0180088)
Supplement: S2 Fig — Receptor density of CD6 expression in unstimulated, Thnp and Th17pol conditions on gated CD8+ lymphocytes is shown as a scatter plot using biotinylated Itolizumab as detection reagent. An increasing trend in CD6 receptor expression is seen in Thnp and Th17 pol conditions over unstimulated cells. Data represents 3 donors. (DOCX) [file pone.0180088.s002.docx]

**S2 Fig.**

**Increased CD6 expression on CD8^+^ lymphocytes**
