# Supplementary material for: T cell activation and differentiation is modulated by a CD6 domain 1 antibody Itolizumab
Source: PLoS One. 2017 Jul 3;12(7):e0180088. doi: 10.1371/journal.pone.0180088 (PMC5495335; doi:10.1371/journal.pone.0180088)
Supplement: S3 Fig — Human PBMCs were left unstimulated or stimulated with soluble anti CD3 0.5 ng/ml (OKT-3) in presence of Iso Ab or Itolizumab at 10 μg/mL for 3 days. Post stimulation, cells were harvested and stained with anti CD6 Ab, MEM98 clone (A) and anti-human IgG, Fc specific (B). In panel A, since the CD6 receptor is occupied with Itolizumab, MEM 98 (commercially available anti CD6 D1) could not bind in Itolizumab treated groups and hence no signal is observed. The positive signal with anti-human IgG, in Itolizumab treated group in panel B suggests that Itolizumab is occupying CD6 receptor on lymphocyte surface. Data is representative of at least 3 independent experiments. (DOCX) [file pone.0180088.s003.docx]

**S3 Fig.**

B

A


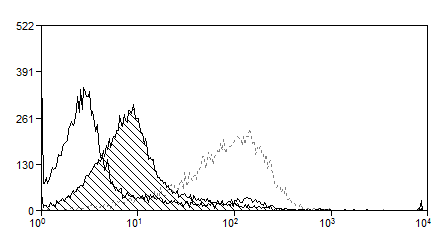

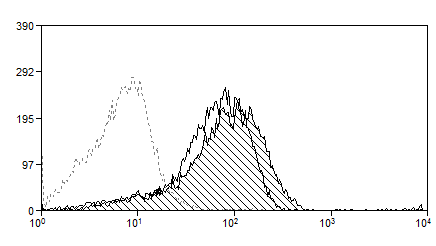


Cell count

CD6

Human IgG

Itolizumab

Iso Ab

Unstimulated

Itolizumab

Iso Ab

Unstimulated

Cell count

**CD6 receptor on lymphocytes is not internalised and is occupied with Itolizumab**
