# Supplementary figures and images for: T cell activation and differentiation is modulated by a CD6 domain 1 antibody Itolizumab
Source: PLoS One. 2017 Jul 3;12(7):e0180088. doi: 10.1371/journal.pone.0180088 (PMC5495335; doi:10.1371/journal.pone.0180088)

**S4 Fig.**


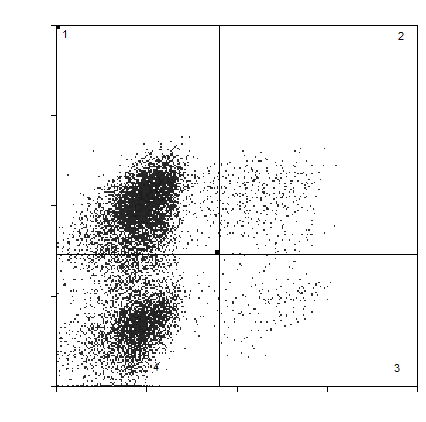


53.5

1.5

41.5

3.5


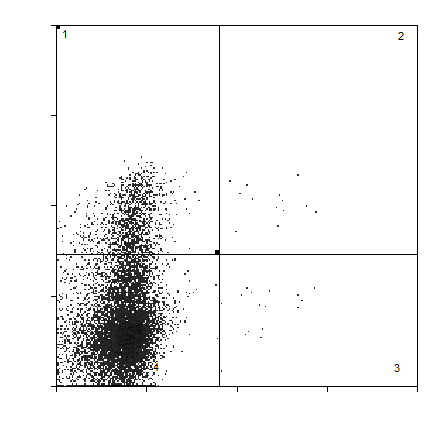


12.1

0.14

87.6

0.12

Iso Ab

Itolizumab

IL17

IFN-γ

**Itolizumab inhibits IFN-γ and IL17-A expression in CD8+lymphocytes**

Supplement: S4 Fig — Human PBMCs were stimulated with anti-CD3 and anti-CD28 beads or soluble anti CD3 0.1 ng/ml (OKT3) and sol anti CD28 (10 ng/ml) in Th17pol conditions in presence of Itolizumab or Iso Ab at 40 μg/mL. On day 6, cells were re-stimulated with PMA-Ionomycin for 5 hours and analyzed for expression of intracellular cytokine IFN-γ and IL-17A. Representative flow cytometry dot plots (gated on lymphocyte scatter and CD8+ lymphocytes) on day 6 are shown in Fig. Percent cells are indicated in the quadrants Itolizumab substantially inhibits IFN-γ and IL-17A expression in CD8+ lymphocytes. Data is representative of 2 independent experiments. (DOCX) [file pone.0180088.s004.docx]
