# Supplementary material for: T cell activation and differentiation is modulated by a CD6 domain 1 antibody Itolizumab
Source: PLoS One. 2017 Jul 3;12(7):e0180088. doi: 10.1371/journal.pone.0180088 (PMC5495335; doi:10.1371/journal.pone.0180088)
Supplement: S5 Fig — (A) Human PBMCs were left unstimulated or stimulated with soluble anti CD3 0.5 ng/ml (OKT-3) in the presence of Iso Ab or Itolizumab at 10 μg/mL for 3 days. Post incubation, cells were harvested and stained with anti CD3, Annexin V and 7-AAD. The % Annexin V positive, 7-AAD negative CD3+T cells has been plotted. The bar graphs show mean±SD from 3 independent experiments. (B) Similar experiment as described in panel A with staining at different time points was done to analyse AICD across days. At each time point, cells were harvested and stained with CD3, Annexin V and 7-AAD. The % Annexin positive, 7-AAD negative CD3+ T cells has been plotted. Data is from one experiment. (DOCX) [file pone.0180088.s005.docx]

**S5 Fig.**

A

B

**Itolizumab does not induce AICD in stimulated PBMC**
