# Supplementary material for: T cell activation and differentiation is modulated by a CD6 domain 1 antibody Itolizumab
Source: PLoS One. 2017 Jul 3;12(7):e0180088. doi: 10.1371/journal.pone.0180088 (PMC5495335; doi:10.1371/journal.pone.0180088)
Supplement: S6 Fig — (A) PBMCs were left unstimulated for 3 days and analysed for expression of intracellular cytokine IFN-γ and IL-17A. Representative flow cytometry dot plots (gated on lymphocyte scatter and CD3+ T-cells) is shown. Percent T-cells are indicated in the quadrants. (B) PBMCs were left unstimulated for 3, 6, 8 and 13 days. Cells were re-stimulated with PMA-Ionomycin for 5 hours and analyzed for expression of intracellular cytokine IFN-γ and IL-17A. Representative flow cytometry dot plots (gated on lymphocyte scatter and CD3+ T cells) across days are shown. Percent T-cells are indicated in the quadrants. In the panels, before gating on lymphocyte gate, total cells were selected and gated to get uniform event count display. (DOCX) [file pone.0180088.s006.docx]

**S6 Fig.**

A


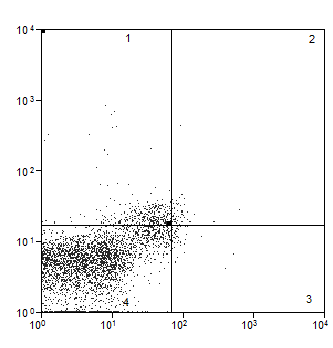


**IL17A**

**IFNγ**

**6.2**

**0.9**

**92.2**

**0.7**

Day3

B


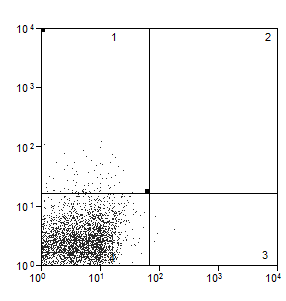

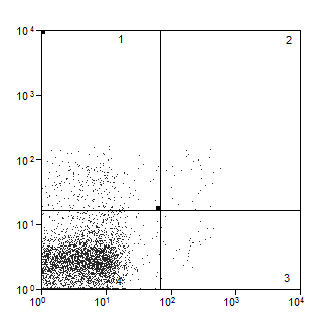

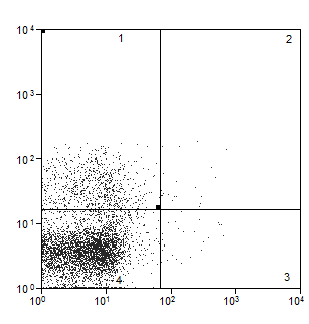

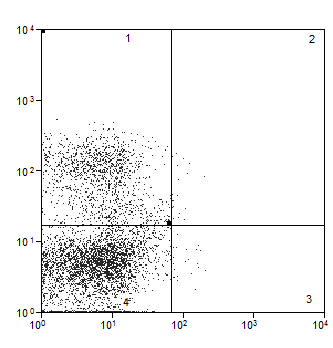


**IL17A**

**IFNγ**

Day13

**1.5**

**0.06**

**98.4**

**0**

**14.5**

**0.4**

**84.8**

**0.3**

**6.0**

**0.4**

**93.1**

**0.4**

**27.3**

**0.3**

**72.0**

**0.3**

Day3

Day6

Day8

**Phenotyping of unstimulated human PBMCs using IL-17 and IFN-γ intracellular cytokine expression across days**
