# Supplementary material for: T cell activation and differentiation is modulated by a CD6 domain 1 antibody Itolizumab
Source: PLoS One. 2017 Jul 3;12(7):e0180088. doi: 10.1371/journal.pone.0180088 (PMC5495335; doi:10.1371/journal.pone.0180088)
Supplement: S7 Fig — PBMCs were left unstimulated or stimulated with soluble anti CD3 0.5 ng/ml (OKT-3) for 3 days / 6 days. Post incubation, cells were harvested and stained with 7-AAD. Panel A shows the representative lymphocyte gate that is put in all experiments. The dead cells (7-AAD positive) seen in green are excluded out of the gate. In panel B, no gate has been applied and positive signal is seen with 7-AAD, indicating the presence of dead cells. In panel C, lymphocyte gate has been applied and the cells do not stain positive for 7-AAD, indicating healthy cell population. Day3 7-AAD staining is a representative of 3 independent experiments and Day6 7-AAD staining is from a single experiment. (DOCX) [file pone.0180088.s007.docx]

**S7 Fig.**

Unstimulated cells

Stimulated cells

Day3

Day6

Day3

Day6

A

B

C

**Lymphocyte gate based on SSC and FSC excludes 7-AAD positive (dead) cells**
