# Supplementary material for: T cell activation and differentiation is modulated by a CD6 domain 1 antibody Itolizumab
Source: PLoS One. 2017 Jul 3;12(7):e0180088. doi: 10.1371/journal.pone.0180088 (PMC5495335; doi:10.1371/journal.pone.0180088)
Supplement: S8 Fig — (A) Undigested Itolizumab (1) and F(ab’)2 fragment of Itolizumab (2) run in non-reducing gel. For undigested Itolizumab band was seen around 150 kDa, while in F(ab’)2 fragment band was seen around 100 kDa. (B) Undigested Itolizumab (2) and F(ab’)2 fragment of Itolizumab (3) run in reducing gel. For undigested Itolizumab two bands were seen around 25 and 50 kDa respectively, while in F(ab’)2 fragment only one band was seen around 25 kDa. (DOCX) [file pone.0180088.s008.docx]

**S8 Fig.**

160

120

100

kDa

1 2

Non reducing condition


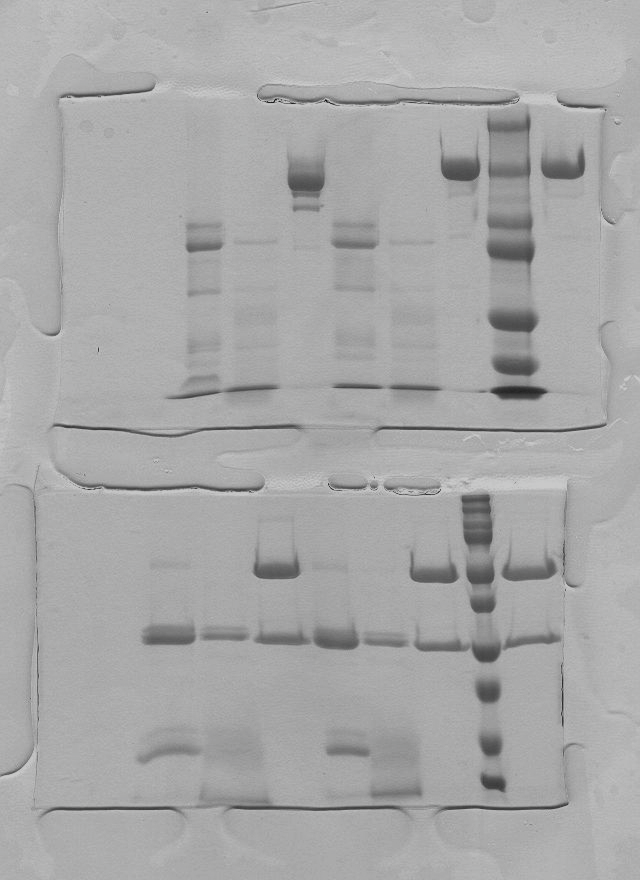


75

55

25

37

1 2

kDa

Reducing condition

1- Undigested Itolizumab

2- F(ab’)2 fragment of Itolizumab

A

B
