# Supplementary material for: T cell activation and differentiation is modulated by a CD6 domain 1 antibody Itolizumab
Source: PLoS One. 2017 Jul 3;12(7):e0180088. doi: 10.1371/journal.pone.0180088 (PMC5495335; doi:10.1371/journal.pone.0180088)
Supplement: S9 Fig — (A) CD6 Western blot of CD6 immune precipitated samples using MEM-98 antibody. (B-F). Quantification (mean±SD) of p-Tyr, Zap70, SLP76, p-SHP1 and p-SHP2 relative intensity as shown in Fig 7A of the manuscript. (B-D) showed a significance difference (p≤ 0.05) while (E-F) showed a trend for reduction with p values 0.1 and 0.2 respectively, when compared between Iso Ab and Itolizumab. (DOCX) [file pone.0180088.s009.docx]

**S9 Fig.**

B

A


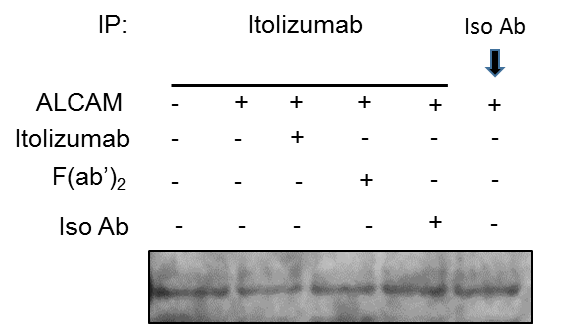

D

C

F

E

**Itolizumab but not its F(ab’)2 fragment inhibits T cell signaling, activation and proliferation**
