# Supplementary material for: T cell activation and differentiation is modulated by a CD6 domain 1 antibody Itolizumab
Source: PLoS One. 2017 Jul 3;12(7):e0180088. doi: 10.1371/journal.pone.0180088 (PMC5495335; doi:10.1371/journal.pone.0180088)
Supplement: S10 Fig — (A) Mouse anti CD6 monoclonal antibody (m CD6D1 mAb) binds to Domain 1 of mouse CD6: Anti CD6 mAb was screened for its binding to plate coated full length, domain-1 and domain-2 of mCD6-Fc by ELISA. mALCAM, hCD6-Fc and anti hCD6 MAb (Itolizumab) were used as controls. Representative graph is shown with mean±SD of two independent experiments. (B) Inhibition of proliferation: Naive mouse splenocytes were added to plate coated with anti CD3 mouse Ab in presence or absence of different concentration of soluble m CD6D1 mAb (2.5–40 μg/ml). Percent Inhibition of proliferation of these splenocytes was calculated with respect to isotype control using Alamar Blue fluorescent dye. Representative graph from three independent experiments is shown. Data is expressed as mean ± SEM. Range of inhibition of m CD6D1 mAb to activated splenocytes is similar to that of Itolizumab on activated T cells. (C) ALCAM binding is independent of m CD6D1 mAb binding to CD6: Mouse CD6-Fc chimera coated plates were incubated with m CD6D1 mAb and/or increasing dose of mouse ALCAM-Fc chimera and binding of m CD6D1 mAb to CD6 was examined. m Iso Ab has a very minimal binding to plate coated mouse CD6-Fc. (DOCX) [file pone.0180088.s010.docx]

**S10 Fig.**

B

A

C

;

**Characterization of m CD6D1 mAb**
