# Supplementary material for: T cell activation and differentiation is modulated by a CD6 domain 1 antibody Itolizumab
Source: PLoS One. 2017 Jul 3;12(7):e0180088. doi: 10.1371/journal.pone.0180088 (PMC5495335; doi:10.1371/journal.pone.0180088)
Supplement: S11 Fig — EAE disease was induced in C57BL/6 normal female mice using MOG35-55 emulsified in CFA and pertussis toxin. Randomized mice were injected with 60 μg in 100 μl volume of either control m Iso Ab (shown in circles) or m CD6D1 mAb (shown as squares) between day 15 and day 27 (arrows indicate days of dosing). 8 animals were used in each group. (A) Clinical scores are represented as Mean ± SEM. (*p≤0.05). (B-C). Splenocytes of these mice were stimulated with soluble MOG antigen peptide (*p≤0.01). (B) Proliferation of these splenocytes were estimated after 96 h using Alamar Blue reagent (C) Supernatants were collected after 48 h and 6 cytokines were analysed using Cytokine bead array (CBA) kit. All cytokines except IL2 and IL-10 showed significant statistical difference using unpaired t-test (*p≤0.05). For IL-10 the p value is 0.07, while for IL-2 only 1 animal had detectable value. (DOCX) [file pone.0180088.s011.docx]

**S11 Fig.**


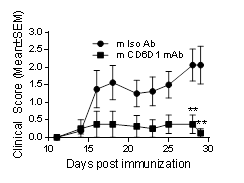


B

A

C

m Iso Ab

m CD6D1 mAb

**m CD6D1 mAb treated EAE-induced mice have hypo-proliferation and lower cytokine release with *ex vivo* MOG treatment**
