# Supplementary material for: T cell activation and differentiation is modulated by a CD6 domain 1 antibody Itolizumab
Source: PLoS One. 2017 Jul 3;12(7):e0180088. doi: 10.1371/journal.pone.0180088 (PMC5495335; doi:10.1371/journal.pone.0180088)
Supplement: S13 Fig — (A) Sequence alignment of Human CD6 SRCR1 (Uniprot ID: P30203) and mouse CD6 SRCR1 (Uniprot ID: Q61003) domains was performed using the Clustal omega (http://www.ebi.ac.uk/Tools/msa/clustalo/). The identity and similarity of the residues was found to be 54.5%, and 64.3% respectively with one gap in the alignment. Secondary structure elements derived from the human CD6 extracellular SRCR domain structure is shown above the sequences. Conservative substitutions are shown in red font and identical residues in white font on a red background. The consensus motif RxE/Q epitope for potential binding to therapeutic mAbs is marked on top with a line. The epitopes identified from point mutants study from Chappell et al., work, the E63 and R77 residues are denoted with # and * respectively. The alignments were generated using the ESPript server (http://espript.ibcp.fr/ESPript/ESPript/). (B) The mouse CD6 SRCR1 domain was modelled using the human CD6 SRCR as template (PDB: 5A2E) using the SWISS-MODEL program (https://swissmodel.expasy.org/). The consensus motif RxE/Q predicted for Itolizumab and UMCD6 mAbs binding, E63 (epitope 1) and R77 (epitope 2) epitopes mapped for binding to MEM98 and MT605 mAbs respectively are marked in the human CD6 SRCR1 domain. The same regions are mapped based on the sequence alignment are marked in the mouse CD6 SRCR1 domain. The numbering of the residues are provided based on the published human CD6 structure (5A2E.PDB). The disordered loop region in both the molecules is shown in grey colour. The N and C regions are marked for orientation of the molecule. All the Figs are prepared using the The PyMOL molecular graphics software (http://www.pymol.org). (DOCX) [file pone.0180088.s013.docx]

**S13 Fig.**

A

**
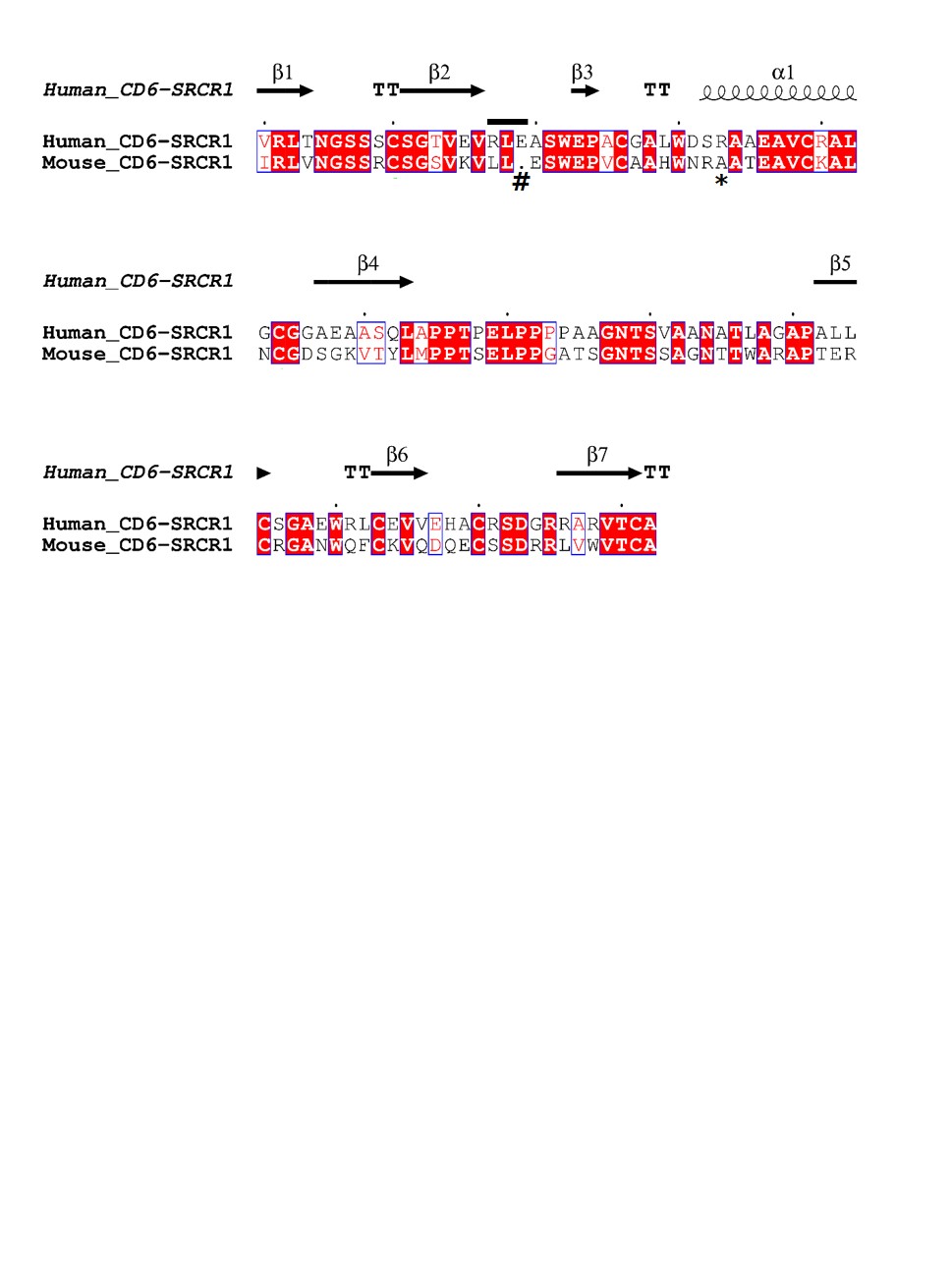
**

B

**
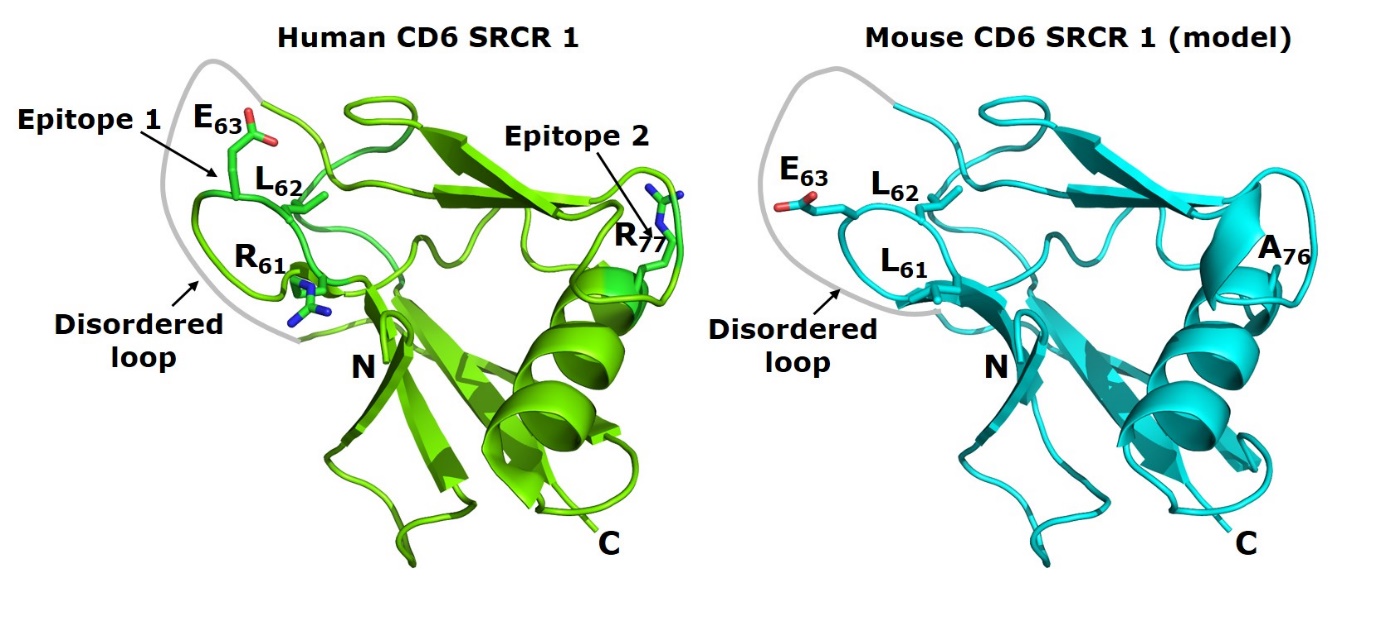
**

**Sequence alignment and three dimensional structural comparison of Human CD6 SRCR1 domain (green) along with the modelled Mouse CD6 SRCR1 domain (cyan).**
