# Supplementary material for: T cell activation and differentiation is modulated by a CD6 domain 1 antibody Itolizumab
Source: PLoS One. 2017 Jul 3;12(7):e0180088. doi: 10.1371/journal.pone.0180088 (PMC5495335; doi:10.1371/journal.pone.0180088)
Supplement: S1 Table — Human PBMCs were left unstimulated or stimulated with anti-CD3, anti-CD28 beads. CD6 expression (using MEM98 as detection reagent) was analyzed on Day 3. The MFI (median fluorescent intensity) of CD6 on unstimulated and stimulated PBMCs gated on CD4+ lymphocytes and on CD4+CD25hi lymphocytes has been tabulated. Data is shown for 3 independent experiments. Representative gating strategy used for gating on CD4+/ CD8+ lymphocytes (gate 1) and CD4+CD25hi / CD8+ CD25hi lymphocytes (gate 2). (DOCX) [file pone.0180088.s014.docx]

**S1 Table. Increase in CD6 MFI on CD4^+^ and CD8^+^ lymphocytes upon activation.**

CD25


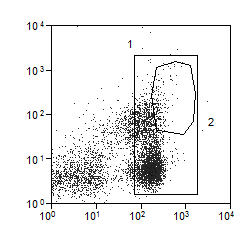

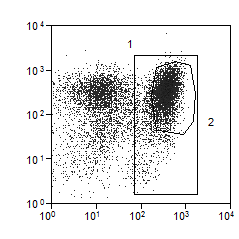


Unstimulated

Stimulated

CD4

| Experiment repeats | **CD6 MFI (median) on Gate 1 (CD4^+^) lymphocytes** | | **CD6 MFI (median) on Gate 2 (CD4^+^CD25^hi^) lymphocytes** |
| --- | --- | --- | --- |
| n | Unstimulated cells | Stimulated cells | Stimulated cells |
| 1 | 113.48 | 168.83 | 188.15 |
| 2 | 73.56 | 131.11 | 140.94 |
| 3 | 49.44 | 73.56 | 81.98 |
|  | **CD6 MFI (median) on Gate 1 (CD8^+^) lymphocytes** | | **CD6 MFI (median) on Gate 2 (CD8^+^CD25^hi^) lymphocytes** |
| n | Unstimulated cells | Stimulated cells | Stimulated cells |
| 1 | 57.13 | 109.45 | 113.48 |
| 2 | 39.81 | 91.37 | 94.73 |
| 3 | 29.82 | 46.00 | 49.44 |
