# Supplementary material for: T cell activation and differentiation is modulated by a CD6 domain 1 antibody Itolizumab
Source: PLoS One. 2017 Jul 3;12(7):e0180088. doi: 10.1371/journal.pone.0180088 (PMC5495335; doi:10.1371/journal.pone.0180088)

**S2 Table. 55 Genes from the current microarray were selected based on previous studies and literature**


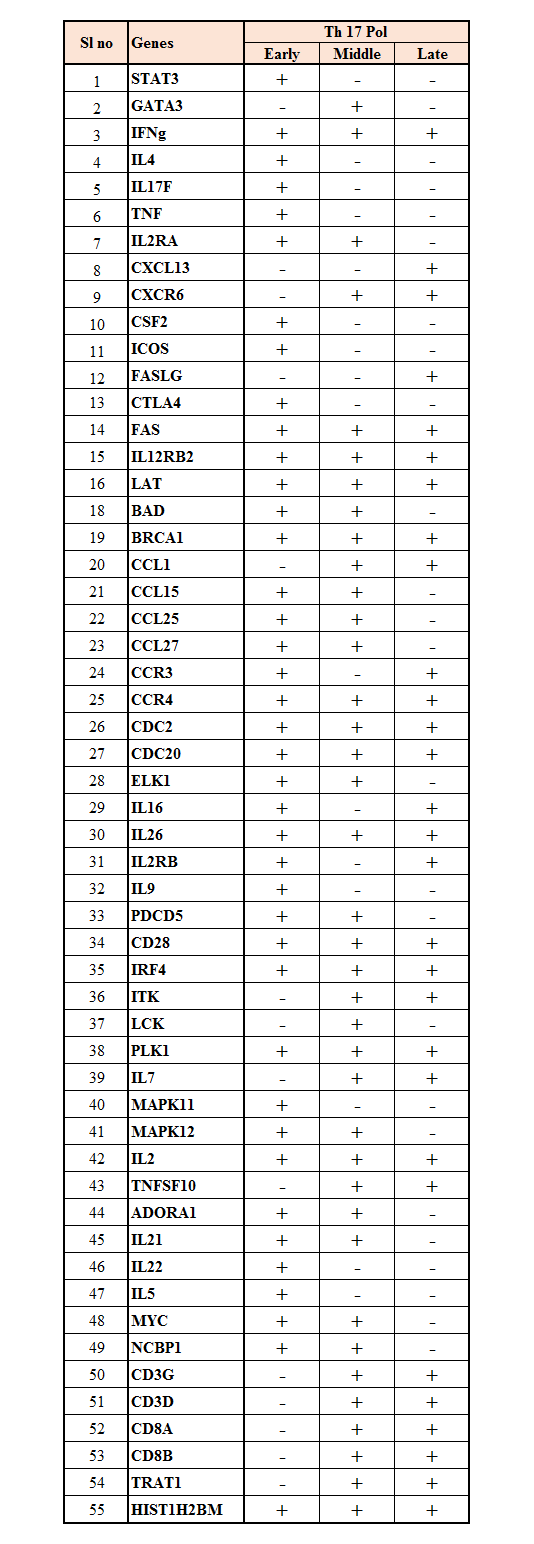

Supplement: S2 Table — (DOCX) [file pone.0180088.s015.docx]
