# Supplementary material for: T cell activation and differentiation is modulated by a CD6 domain 1 antibody Itolizumab
Source: PLoS One. 2017 Jul 3;12(7):e0180088. doi: 10.1371/journal.pone.0180088 (PMC5495335; doi:10.1371/journal.pone.0180088)
Supplement: S3 Table — (DOCX) [file pone.0180088.s016.docx]

**S3 Table. Binding kinetics of m CD6D1 mAb, Itolizumab full length and its F(ab’)2 fragment to their respective full length CD6 ligand by surface plasmon resonance method**

| **Name of the antibody** | **K_D_ (nM)** |
| --- | --- |
| m CD6D1 mAb | 1.77 |
| Itolizumab | 1.33 |
| F(ab’)2 fragment of Itolizumab | 1.18 |
