# Supplementary material for: T cell activation and differentiation is modulated by a CD6 domain 1 antibody Itolizumab
Source: PLoS One. 2017 Jul 3;12(7):e0180088. doi: 10.1371/journal.pone.0180088 (PMC5495335; doi:10.1371/journal.pone.0180088)
Supplement: S4 Table — (DOCX) [file pone.0180088.s017.docx]

**S4 Table. Percentage reduction in EAE clinical score during treatment over the control in 6 independent EAE studies**

| **Study number** | **Max % reduction Observed during treatment** |
| --- | --- |
| 1 | 100 |
| 2 | 43.64 |
| 3 | 44.23 |
| 4 | 69.08 |
| 5 | 52.08 |
| 6 | 55.23 |
| Mean | 61.80 |
| SD | 23.70 |
| Max % reduction | 100 |
| Min % reduction | 43.64 |
